# Divergent androgen regulation of unfolded protein response pathways drives prostate cancer

**DOI:** 10.15252/emmm.201404509

**Published:** 2015-04-11

**Authors:** Xia Sheng, Yke Jildouw Arnoldussen, Margrethe Storm, Martina Tesikova, Hatice Zeynep Nenseth, Sen Zhao, Ladan Fazli, Paul Rennie, Bjørn Risberg, Håkon Wæhre, Håvard Danielsen, Ian G Mills, Yang Jin, Gökhan Hotamisligil, Fahri Saatcioglu

**Affiliations:** 1 Department of Biosciences, University of OsloOslo, Norway; 2 The Vancouver Prostate CentreVancouver, BC, Canada; 3 Institute for Cancer Genetics and Informatics, Oslo University HospitalOslo, Norway; 4 Division of Pathology, Oslo University HospitalOslo, Norway; 5 Division of Surgery, Oslo University HospitalOslo, Norway; 6 Center for Cancer Biomedicine, University of OsloOslo, Norway; 7 Department of Informatics, University of OsloOslo, Norway; 8 The Centre for Molecular Medicine Norway, University of OsloOslo, Norway; 9 Department of Urology, Oslo University HospitalOslo, Norway; 10 Department of Cancer Prevention, Institute of Cancer Research, Radium HospitalOslo, Norway; 11 Department of Genetics and Complex Diseases, Harvard School of Public Health, Harvard UniversityBoston, MA, USA

**Keywords:** androgen receptor, androgens, ER stress, prostate cancer, UPR

## Abstract

The unfolded protein response (UPR) is a homeostatic mechanism to maintain endoplasmic reticulum (ER) function. The UPR is activated by various physiological conditions as well as in disease states, such as cancer. As androgens regulate secretion and development of the normal prostate and drive prostate cancer (PCa) growth, they may affect UPR pathways. Here, we show that the canonical UPR pathways are directly and divergently regulated by androgens in PCa cells, through the androgen receptor (AR), which is critical for PCa survival. AR bound to gene regulatory sites and activated the IRE1α branch, but simultaneously inhibited PERK signaling. Inhibition of the IRE1α arm profoundly reduced PCa cell growth *in vitro* as well as tumor formation in preclinical models of PCa *in vivo*. Consistently, AR and UPR gene expression were correlated in human PCa, and spliced XBP-1 expression was significantly upregulated in cancer compared with normal prostate. These data establish a genetic switch orchestrated by AR that divergently regulates the UPR pathways and suggest that targeting IRE1α signaling may have therapeutic utility in PCa.

## Introduction

The endoplasmic reticulum (ER) is an essential organelle which regulates protein folding and secretion and impacts key functions in the cell, such as lipid biosynthesis, and calcium homeostasis (Hetz, 2012). Different physiological and pathological conditions interfere with the protein folding capacity of the ER, which leads to the accumulation of unfolded or misfolded proteins, named ER stress (for a review, see Tabas & Ron, 2011). In an attempt to cope with the stress, several intracellular signal transduction pathways, collectively termed the unfolded protein response (UPR), are activated. The UPR signaling aims to increase the protein folding capacity in the ER lumen, thus decreasing the unfolded protein load on the cell. If the UPR is unsuccessful, however, apoptotic pathways are activated and cell death results.

The UPR is mediated by at least three well-conserved stress sensors that are ER-localized transmembrane receptors: pancreatic ER kinase-like ER kinase (PERK), activating transcription factor 6 (ATF6), and inositol-requiring kinase 1 (IRE1) (Tabas & Ron, 2011). In the canonical model, in unstressed cells, these proteins are held in an inactive state by protein chaperones, which inhibit their activity. Upon accumulation of unfolded or misfolded proteins, chaperones dissociate from the transmembrane receptors and bind to the unfolded proteins in the lumen of the ER allowing IRE1α and PERK oligomerization in the ER membrane, and translocation of ATF6α to the Golgi where it is cleaved into an active transcription factor. This then leads to specific gene expression and signaling which in turn orchestrates adaptation to ER stress.

Endoplasmic reticulum stress has been linked to many chronic diseases, such as diabetes, neurodegeneration, various cancers, and proinflammatory conditions (for reviews, see Hotamisligil, 2010; Clarke *et al*, 2014). In cancer, a wide range of cytotoxic conditions such as hypoxia, pH changes, and nutrient deprivation trigger the activation of the UPR to help the cancer cells to cope with the stress. Thus, the ER stress response in this setting could be a cytoprotective response with an important role in tumor growth, especially in tumors arising from active secretory cells, such as the case in multiple myeloma (for a review, see Tsai & Weissman, 2010). For example, the IRE1α-X-box-binding protein 1 (XBP-1) pathway has been shown to promote tumor growth in xenograft models (Romero-Ramirez *et al*, 2004) and loss of XBP-1 sensitized cells to death from oxidative stress (Liu *et al*, 2009). Transgenic mice studies have shown that XBP-1 splicing occurs during primary tumor growth in a number of breast cancer models (Spiotto *et al*, 2010). However, under certain conditions, activation of the IRE1α or PERK pathways may lead to apoptosis, for example by activating c-Jun N-terminal kinase (JNK), and thereby inhibiting tumor growth (for a review, see Clarke *et al*, 2014). Furthermore, UPR can impact autophagy and mitophagy, two processes that can impart cancer cells survival benefit (Kroemer *et al*, 2010; Maes & Agostinis, 2014). Thus, the role of UPR in cancer cells is paradoxical: it is involved in the adaptive response of tumor cells, but also can initiate apoptotic cell death (for a review, see Liu & Ye, 2011; Vandewynckel *et al*, 2013; Clarke *et al*, 2014).

There is limited information on ER stress and UPR pathways in PCa cells to date. Global gene expression profiling experiments in androgen-treated PCa cells have shown changes in the expression of some ER stress-associated genes, such as N-myc downstream-regulated gene 1 protein (*NDRG1*), protein disulfide isomerase-related protein (*PDIR*), homocysteine-responsive ER-resident ubiquitin-like domain member 1 protein (*HERPUD1*), and oxygen-regulated protein 150 (*ORP150*) (Segawa *et al*, 2002). In tumor models, gene expression profiling indicated downregulation of the UPR branches in high-grade PIN in *Nkx3.1*:*Pten* mutant mice, a mouse model of PCa. Expression of some ER-associated molecules, such as HERPUD1 and NDRG1, was reduced in PCa samples from patients (Segawa *et al*, 2002), and GRP78 expression levels were associated with greater risk of PCa recurrence and worse survival (Pootrakul *et al*, 2006; Daneshmand *et al*, 2007).

PCa cells are highly secretory and are regulated by hormonal signals, in particular androgen signaling, via the androgen receptor (AR), which is important in the initiation and progression of PCa (for a review, see Bluemn & Nelson, 2012). We thus postulated that PCa cells may have developed ways to engage the ER adaptive responses and hormonally regulate UPR to sustain normal tissue integrity which may also support prostate tumorigenesis. Here, we show that androgens induce a unique UPR profile in PCa cells by activating the IRE1α branch, but coordinately inhibit PERK signaling, to regulate growth and survival of PCa *in vitro* and *in vivo*. These data may have translational implications.

## Results

### AR and UPR gene expression are correlated in prostate cancer

**Figure 1:**
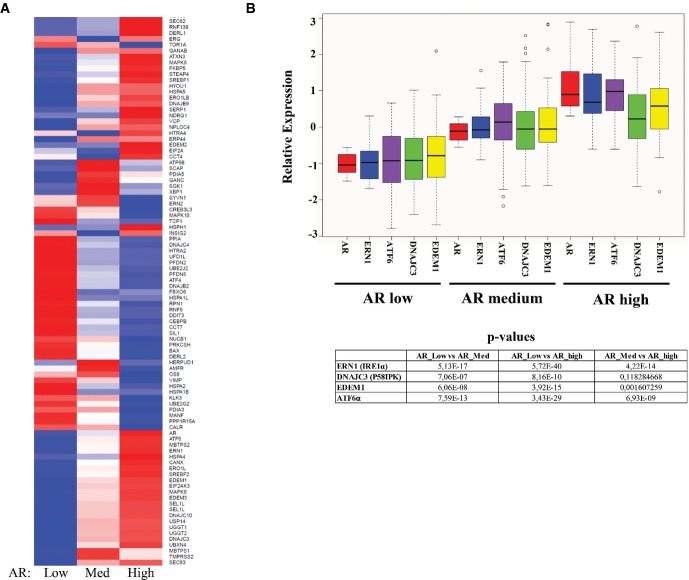
Correlation of AR and UPR gene expression in prostate cancer cohorts

Given the role of androgens in PCa progression and the secretory function of the prostate that would increase burden on ER, we hypothesized that UPR signaling may be affected by AR signaling. To assess this, we investigated the possible concordance between AR and UPR gene expression in a gene expression data set from 190 human PCa tumors. AR expression in the PCa tumors was significantly correlated with UPR gene expression (Supplementary Fig S1 and Supplementary Table S1). The tumors were then stratified into three groups according to AR status, that is AR_Low_ (*n* = 60), AR_medium_ (*n* = 70), and AR_high_ (*n* = 60), and assessed for UPR gene expression. As shown in Fig1A, stratified AR levels correlated with UPR gene expression (Supplementary Table S2). The expression profiles of prominent UPR genes, including ERN1 (IRE1), ER degradation-enhancing alpha-mannosidase-like 1 (EDEM1), ATF6 and DNAJC3 (or 58 kDa interferon-induced protein kinase, P58IPK), are presented separately for ease of evaluation (Fig1B). These data were validated using an independent patient cohort (Supplementary Table S3), suggesting that AR and UPR gene expression are linked in PCa.

Possible correlation between AR- and UPR-associated gene expression was assessed in the global gene expression data available in the TCGA Prostate Adenocarcinoma cohort (*n* = 190) (http://www.cbioportal.org/public-portal/index.do). Tumors were stratified according to AR status into three groups, that is AR_low_ (*n* = 60), AR_medium_ (*n* = 70), and AR_high_ (*n* = 60). The levels of UPR gene expression in the three groups were compared using Pearson's correlation analysis by the R software and presented as a heatmap. There were significant differences between the three groups (Supplementary Table S2).

The expression profiles of some prominent UPR genes from the data in (A), including ERN1 (IRE1), EDEM1, ATF6, and DNAJC3 (P58IPK), are presented. *P*-values of the different genes are given.

### Androgens activate the IRE1α branch of the UPR *in vitro* and *in vivo*

**Figure 2:**
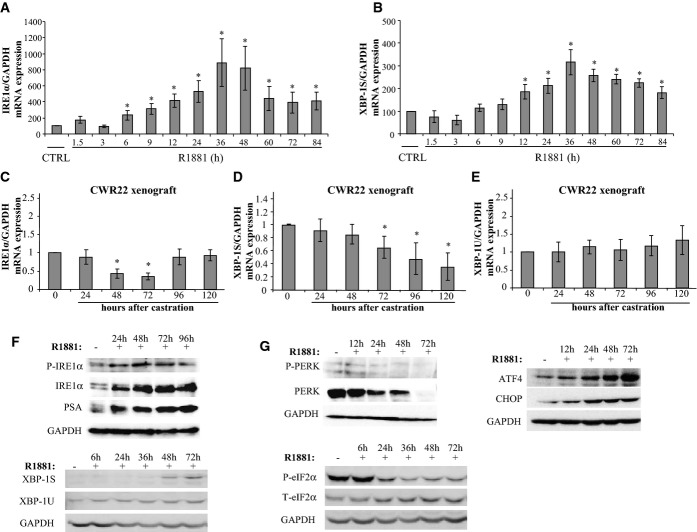
Androgens divergently regulate the UPR arms LNCaP cells were cultured and treated with R1881 for the indicated times.

We next assessed whether androgens affect UPR gene expression in LNCaP PCa cells. *IRE1α* expression was significantly increased in a time-dependent manner upon androgen administration (Fig2A). Consistently, the expression of the principal IRE1α target, *XBP-1S,* was significantly increased in a similar manner (Fig2B). In contrast, there was a very marginal, but significant change in the levels of unspliced *XBP-1* expression (Supplementary Fig S2A). Furthermore, the expression of several established XBP-1S target genes, such as ER-localized DnaJ 4 (*ERdj4*), *P58IPK*, ribosome-associated membrane protein 4 (*RAMP4*), and *EDEM1*, was robustly increased in response to androgen treatment (Supplementary Fig S2B–E). We then studied possible androgen regulation of the IRE1α branch in a preclinical model of human PCa, CWR22. In response to androgen withdrawal by castration, CWR22 tumors regress due to a decrease in cell growth and an increase in apoptosis, similar to the *in situ* disease (Wainstein *et al*, 1994). *IRE1α* expression significantly decreased upon castration up to 72 h followed by an increase back to basal levels by 120 h (Fig2C). *XBP-1S* expression significantly decreased after 72 h reaching approximately 40% of basal levels at 120 h (Fig2D), whereas *XBP-1U* expression was not affected (Fig2E). Consistently, the IRE1α pathway was also activated at the protein level with increases in phosphorylated IRE1α, total IRE1α and XBP-1S levels in LNCaP cells upon androgen treatment (Fig2F).

A, B mRNA expression levels in LNCaP cells for the indicated genes were investigated using quantitative PCR (qPCR). Controls were treated with vehicle for 84 h and set to 100. Data represent the mean of three independent experiments in triplicate, and bars represent SE. *P*-values ranged between 1.66 × 10^−5^ and 0.025, and IRE1α expression in R1881 48 h was *P = 0.013 with respect to vehicle-treated cells using unpaired Student's *t*-test.

C–E *IRE1α*, *XBP-1S*, and *XBP-1U* mRNA in CWR22 xenografts grown in nude mice and collected at the indicated times after castration. The value at *t* = 0 was set to 1. Columns represent the mean of at least three independent tumors for each time point, and bars represent SE. *P*-values ranged between 5.7  ×  10^−10^ and 0.0002. IRE1α expression at 48 h post-castration was 3.17 × 10^−6^ with respect to *t* = 0 using unpaired Student's *t*-test.

F, G Protein expression in LNCaP cells upon treatment with R1881 for the indicated times by Western blot analysis. Data presented are representative of three independent experiments.

Source data are available online for this figure.

### Androgens differentially regulate the three canonical UPR pathways

We then assessed possible androgen effects on the PERK pathway. PERK activation results in eIF2α phosphorylation which inhibits translation, thus alleviating ER stress by decreasing misfolded protein overload. Both total and phosphorylated PERK levels were significantly decreased in LNCaP cells upon androgen treatment (Fig2G). Consistently, p-eIF2α levels rapidly decreased upon androgen exposure confirming inhibition of the PERK pathway (Fig2G). In addition to general inhibition of protein synthesis, eIF2α phosphorylation simultaneously promotes the translation of a subset of UPR target proteins such as ATF4 (Holcik & Sonenberg, 2005). Whereas *ATF4* mRNA expression was not affected (Supplementary Fig S3A), ATF4 protein levels were increased (Fig2F). In addition, expression of CHOP, a downstream target of ATF4, was significantly decreased upon androgen treatment (Supplementary Fig S3B) at the mRNA level, but its protein levels increased in response to androgen treatment (Fig2G). Altogether, these data indicate that androgens selectively activate the adaptive IRE1α arm of the UPR, while simultaneously inhibiting the PERK branch. Supporting this, similar data were obtained in VCaP cells, another androgen-responsive cell line (Supplementary Fig S4A). LNCaP cells treated with the natural androgen dihydrotestosterone (DHT) induced a similar UPR response as R1881 with an increase in IRE1α and a downregulation in p-eIF2α expression (Supplementary Fig S4B), confirming that the divergent UPR response to androgens is physiological.

To determine whether androgens may also affect the third canonical UPR pathway, we investigated the targets of the ATF6α branch. The reagents available are at present limited to assay for the activation of this pathway in human cells. However, as shown above (Supplementary Fig S2A), the ATF6α target *XBP-1U* expression was only slightly increased upon androgen treatment. Similarly, expression of another ATF6α target gene, *GRP78,* was only modestly (approximately 2-fold) increased by androgens (Supplementary Fig S3C). These data suggest that androgens may activate the ATF6α pathway, but to a significantly lesser degree compared to the IRE1α arm.

### IRE1α signaling inhibits apoptosis in prostate cancer cells

One target of IRE1α is c-Jun N-terminal Kinase (JNK) (Urano *et al*, 2000; Nishitoh *et al*, 2002). Since IRE1α-XBP-1S signaling is generally involved in proliferative effects, whereas JNK induces apoptosis in PCa cells (Lorenzo & Saatcioglu, 2008), we determined androgen effects on JNK activation. UV-induced JNK activation was significantly reduced in response to androgen stimulation (Supplementary Fig S4C). These data are consistent with previous findings showing that androgens block JNK activation in PCa cells, triggered by UPR inducers, such as thapsigargin (e.g., Lorenzo & Saatcioglu, 2008).

We next determined whether IRE1α knockdown may have an effect on PCa cell viability. As shown in Supplementary Fig S5A, there was a significant increase in apoptosis in LNCaP cells upon IRE1α knockdown which coincided with cleavage of caspase-3 (Supplementary Fig S5B). XBP-1 knockdown also increased caspase-3 cleavage, suggesting that the whole IRE1α-XBP-1 signaling is involved in cell viability in PCa cells. Together with the results from above, these data show that androgens activate the proliferative IRE1α signaling and simultaneously inhibit proapoptotic JNK signaling.

### AR knockdown differentially affects UPR gene expression

**Figure 3:**
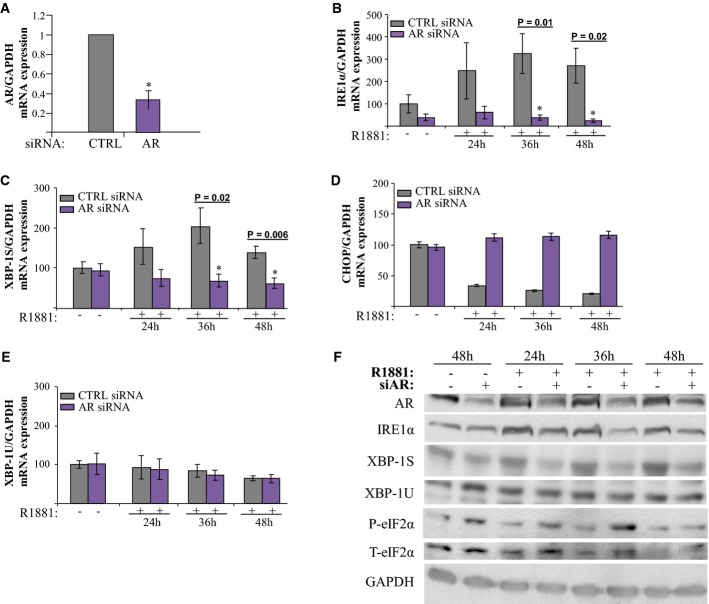
AR knockdown differentially influences transcription of the different UPR members After starvation, LNCaP cells were transfected with control (CTRL) siRNA or AR siRNA. Cells were then treated with R1881 for the indicated times. Controls were treated with vehicle for 48 h.
A Expression level of AR mRNA upon siRNA treatment for 48 h assessed by qPCR in LNCaP cells. Expression in cells transfected with CTRL siRNA was set to 1. Bars represent SE with *P = 0.001 indicating significant difference between AR siRNA- and control siRNA-transfected cells using paired Student's *t*-test.

We then investigated whether the androgen effects on UPR signaling require AR. siRNA-mediated knockdown of AR in LNCaP cells resulted in 60–70% reduction in AR levels (Fig3A), which completely blocked R1881-induced *IRE1α* expression compared to cells treated with control siRNA (Fig3B). Similarly, AR knockdown prevented androgen-induced *XBP-1S* expression (Fig3C), whereas *XBP-1U* expression was not affected (Fig3E). In contrast, AR knockdown increased *CHOP* expression (Fig3D) without affecting *ATF4* levels (Supplementary Fig S6E). Expression of other XBP-1S targets, such as *P58IPK*, *EDEM1, RAMP4*, and *ERdj4,* was also significantly decreased upon AR knockdown (Supplementary Fig S6A–D) underscoring the importance of AR for IRE1α signaling in PCa cells. Similar results were obtained at the protein level (Fig3F, quantification is shown in Supplementary Fig S6F). Taken together, these data show that AR is required for selective androgen regulation of the canonical UPR pathways.

B–E Same as in (A), but mRNA expression levels of the indicated UPR genes were determined by qPCR at indicated time points after R1881 stimulation. Expression in cells transfected with CTRL siRNA was set to 100. Bars represent SE. *P*-values are shown indicating significant difference between AR siRNA- and control siRNA-transfected cells using unpaired Student's *t*-test.

F Expression of the indicated proteins under conditions indicated on the top label was determined by Western blot analysis. Representative blots for three independent experiments are shown.

Source data are available online for this figure.

### Direct AR binding to UPR gene regulatory sequences

**Figure 4:**
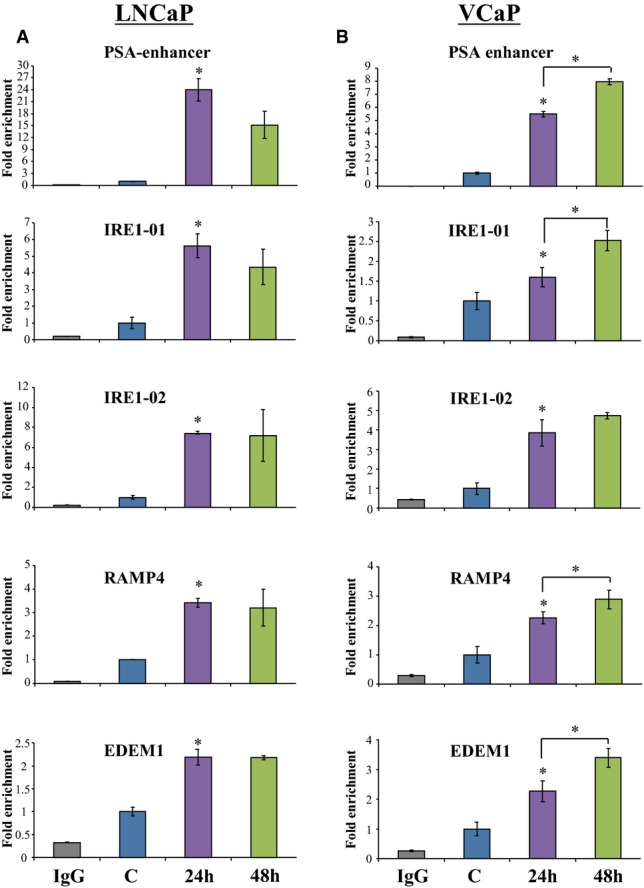
AR directly binds in the vicinity of different UPR genes

The data presented above indicated that AR may directly bind to regulatory sites in UPR genes. To assess this, we examined a data set from chromatin immunoprecipitation sequencing (ChIP-seq) experiments with AR in LNCaP and VCaP cells (Massie *et al*, 2011) which suggested that AR directly binds to several of the UPR-associated genes. To validate these observations, we performed individual ChIP experiments. AR efficiently loaded on to its classical target (Brookes *et al*, 1998) in the PSA enhancer upon androgen treatment in LNCaP cells (Fig4A). Two sites predicted by ChIP-Seq data for *IRE1α* showed approximately 6- and 8-fold enrichment of AR binding upon androgen treatment (Fig4A). In addition, XBP-1S target genes *RAMP4* and *EDEM1* had a 2.5- to 4-fold increase in AR binding upon androgen treatment (Fig4A). Similar results were obtained in VCaP cells (Fig4B). These data show that AR directly binds to regulatory sites in the vicinity of genes involved in the IRE1α pathway and regulate their expression.

A, B LNCaP (A) or VCaP cells (B) were cultured and treated with vehicle (C) for 48 h or R1881 for 24 and 48 h. The cells were then fixed, and ChIP assay was performed as described in Materials and Methods using AR antibody. The data shown are representative of one experiment in duplicate. Error bars represent SE. *P < 0.01 for LNCaP and *P < 0.04 for VCaP indicate significant difference between C (control) and R1881 using unpaired Student's *t*-test.

### IRE1α or XBP-1 loss inhibits prostate cancer cell growth both *in vitro* and *in vivo*

**Figure 5:**
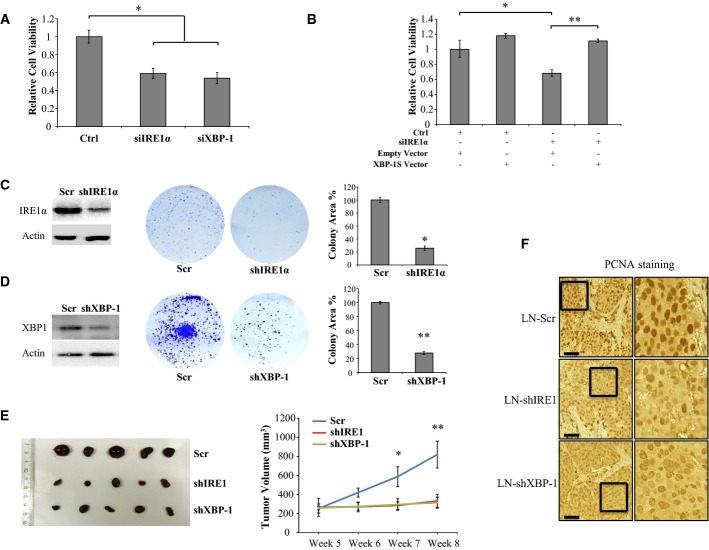
IRE1α and XBP-1 are proliferative factors in PCa cells both *in vitro* and *in vivo*

Since AR signaling is an established proliferative pathway in PCa cells, we explored whether IRE1α signaling may affect PCa cell growth. Knockdown of IRE1α or XBP-1 led to a significant decrease in LNCaP cell proliferation (Fig5A). Ectopic expression of XBP-1S in IRE1α knockdown cells restored proliferation back to the level of control cells (Fig5B) showing that the effects of IRE1α in PCa cells are mediated through XBP-1S. These findings support the data from above that IRE1α pathway, through XBP-1S, increases proliferation in PCa cells.

A Knockdown of IRE1α or XBP-1 leads to a decrease in cell survival. LNCaP cells were transfected with siRNA targeting either IRE1α or XBP-1 (5 nM) and starved in 2% CT-FCS medium for 3 days before cell viability was measured using the CCK-8 assay. The graph is representative of one experiment in triplicate and was repeated three times with similar results. Error bars represent SD with *P = 6.6 × 10^−5^ and 4.5 × 10^−5^ for comparison between Ctrl and siRNA against IRE1α and XBP-1, respectively, using paired Student's *t*-test.

B XBP-1 rescues the growth defect of siIRE1α-transfected LNCaP cells. LNCaP cells were transfected with 5 nM of indicated siRNA using Lipofectamine RNAiMax reagent. One day after siRNA transfection, the cells were transfected with either vector control (Empty) or Flag-XBP-1S (XBP-1S). Three days after transfection, cells were harvested for Western analysis or cultured for three more days before being applied to cell proliferation assay using the CCK-8 reagent. The data are representative of two experiments in triplicate. Error bars represent SE. *P = 0.02, **P = 8.54 × 10^−7^ using paired Student's *t*-test.

C, D IRE1α and XBP-1 knockdown inhibits clonogenic capacity of LNCaP cells. Control LN-Scr (Scr), LN-shIRE1 (shIRE1), or LN-shXBP1 (shXBP-1) cells were cultured for 3 weeks. The colonies formed were stained with crystal violet and photographed. The extent of IRE1α and XBP-1 knockdown was determined by Western blot analysis. The area covered by colonies was quantified using the Gene Tools software (SynGene). The data are representative of three experiments in triplicate. Error bars represent SEM. *P = 4.38 × 10^−7^ and **P = 3.39 × 10^−20^ using paired Student's *t*-test.

E Growth analysis of xenografted LNCaP tumors in nude mice. LNCaP cells expressing shRNA against IRE1α (LN-shIRE1), XBP1 (LN-shXBP-1), or control shRNA (LN-Scr) were subcutaneously implanted into both flanks of male nude mice (6 mice per group). Tumor size was measured at the indicated time points. Representative pictures of the tumors at harvest are shown. Error bars indicate SEM. *P = 0.03 for shIRE1 at week 7, *P* = 0.02 for shXBP-1 at week 7, **P = 0.01 for both shIRE1 and shXBP-1 at week 8 using unpaired Student's *t*-test.

F PCNA immunostaining in tumors from animals bearing LN-shIRE1, LN-shXBP-1, or LN-Scr tumors. Scale bars: 100 μm.

To further explore the nature of IRE1α signaling in PCa, we established LNCaP cell lines stably expressing short hairpin RNAs (shRNAs) directed against IRE1α and XBP-1 using lentiviral gene delivery (Fig5C and D). The IRE1α-depleted cells (LN-shIRE1) had significantly reduced proliferation compared with control cells (LN-shScr) (Fig5C and Supplementary Fig S7A–C); similar results were obtained upon XBP-1 depletion (Fig5D) consistent with transient knockdown experiments (Fig5A). The reduction in growth in either cell line was rescued, at least in part, by androgen treatment, and the rescue was almost complete for LN-shXBP-1 cells upon long-term androgen treatment (Supplementary Fig S7A and B). The levels of AR or its responsiveness to R1881 were essentially the same in the different cell lines (Supplementary Fig S7D). Taken together, these data indicate that the androgen-mediated induction of IRE1α and XBP-1S confers a survival advantage to PCa cells. However, IRE1α depletion did not synergize with the anti-androgen MDV3100 on LNCaP cell growth inhibition, suggesting that other factors may be involved in this process (Supplementary Fig S7E).

To check the *in vivo* validity of these findings, IRE1α and XBP-1 knockdown lines were used in xenograft experiments in nude mice. Whereas all cell lines formed tumors and the control LN-Scr tumors continued to grow throughout the experiment, LN-shIRE1 and LN-shXBP-1 tumors essentially stopped growing at 4–5 weeks and by 8 weeks were only approximately 40% in size compared with control tumors (Fig5E). In agreement with this, there was a decrease in staining of the proliferative marker PCNA in tumors derived from LN-shIRE1 and LN-shXBP-1 cells compared to control tumors (Fig5F). These data show that the IRE1α/XBP-1 pathway is essential for PCa tumor growth *in vitro* and *in vivo*.

### A small molecule inhibitor of IRE1α blocks prostate cancer growth

**Figure 6:**
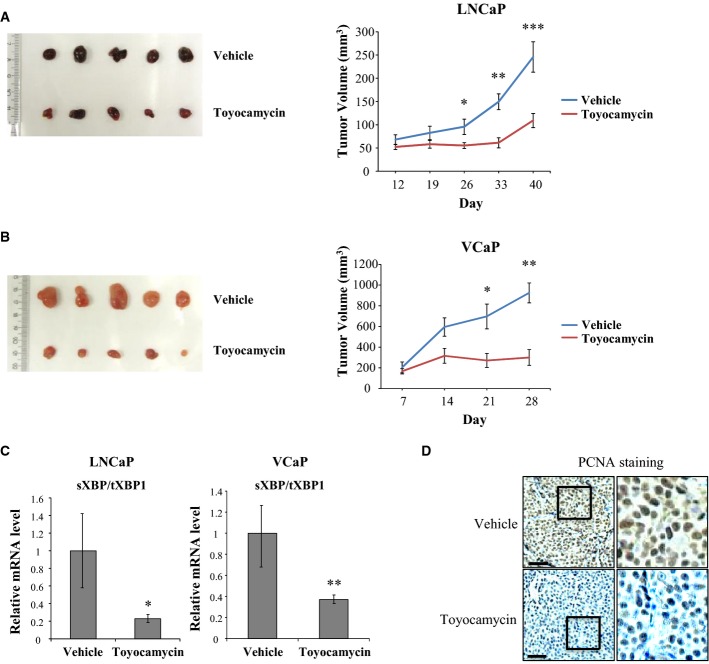
A small molecule IRE1α inhibitor interferes with prostate cancer cell growth *in vivo* LNCaP xenografts were grown in nude mice until palpable. Mice were then intraperitoneally injected with 0.5 mg/kg toyocamycin or saline (Vehicle) (tumor numbers: *n* = 15, or *n* = 10, respectively) twice weekly.
Tumor sizes were measured weekly with calipers. Error bars indicate SEM. *P = 0.04; **P = 0.0004; ***P = 0.002 using unpaired Student's *t*-test.

The data presented above showed that IRE1α signaling is critical for PCa cell viability and growth. We therefore evaluated the possibility that specific pharmacologic inhibition of IRE1α can interfere with PCa growth. To that end, we used a recently identified inhibitor of IRE1α, toyocamycin (Ri *et al*, 2012), and assessed its ability to inhibit IRE1α signaling and PCa growth *in vitro* and *in vivo*. Consistent with findings in other cell lines (Ri *et al*, 2012), toyocamycin inhibited XBP-1 splicing in LNCaP cells confirming IRE1α inhibition (Supplementary Fig S8A). Toyocamycin treatment inhibited LNCaP cell growth in a dose-dependent manner (Supplementary Fig S8B). Consistently, when LNCaP cells were grown as xenografts in nude mice, tumor growth in mice injected with toyocamycin was significantly slower compared to mice receiving vehicle alone (Fig6A). Similar results were obtained in experiments using VCaP cells (Fig6B). The level of spliced XBP-1 was significantly lower in tumors treated with toyocamycin compared to control tumors, confirming that the observed results are caused by inhibition of IRE1α (Fig6C). Consistent with reduced proliferation in response to toyocamycin, PCNA expression in tumors significantly decreased upon toyocamycin treatment (Fig6D). Taken together, these results show that specific pharmacologic targeting of IRE1α can be a potential therapeutic strategy for PCa.

Same procedure was repeated on VCaP xenografts, with similar findings as in LNCaP xenografts (tumor numbers: *n* = 9, *n* = 11). Error bars indicate SEM. *P = 0.02 and **P = 0.0006 using unpaired Student's *t*-test.

*XBP-1S* mRNA levels in LNCaP xenografts from animals treated with toyocamycin or vehicle. Tumors were harvested, RNA-extracted, and qPCR performed. Error bars indicate SEM. *P = 0.04 for LNCaP and **P = 0.02 for VCaP using unpaired Student's *t*-test.

PCNA staining in tumors from animals treated with either toyocamycin or saline. Scale bar: 100 μm.

### XBP-1S expression in human prostate cancer

**Figure 7:**
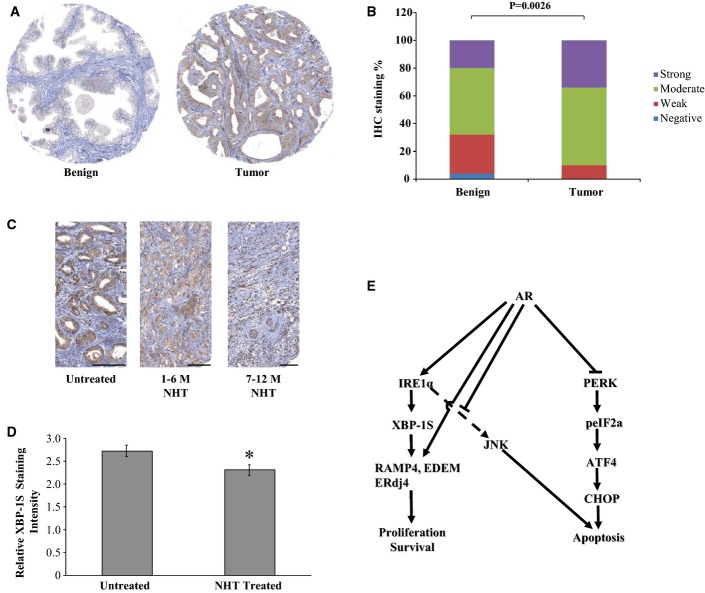
XBP-1S expression in clinical prostate cancer specimens XBP-1S expression was determined by IHC in two different cohorts of human prostatectomy samples.
Representative pictures of benign and tumor samples.

To evaluate the potential application of our findings to human PCa further, we examined the expression of XBP-1S by immunohistochemical analysis on human radical prostatectomy specimens (the available antisera for IRE1α did not function in this analysis). XBP-1S protein was expressed in the benign prostate, predominantly in epithelial cells, and its expression was significantly increased in PCa specimens compared to normal tissue controls (Fig7A and B and Supplementary Table S4). In addition, XBP-1S expression was reduced following neoadjuvant hormone therapy and remained low in patients that responded to therapy, indicating that XBP-1S is regulated by AR *in vivo* and may contribute to castrate-resistant disease (Fig7C and D). These data show that the activity of the IRE1α arm of the UPR is deregulated in human PCa and may have a role in disease progression.

Tissue microarrays (Wang *et al*, 2010) containing 25 benign and 283 tumor samples were stained with a XBP-1S specific antiserum and scored by a pathologist. The *P*-value indicates the difference between XBP-1S staining (strong and moderate) in normal vs cancer cells using Mann–Whitney test.

XBP-1S expression was determined by IHC of a neoadjuvant hormone therapy (NHT) tissue microarray containing samples from hormone naïve (untreated) (*n* = 25), patients that received NHT for 1–6 months (*n* = 33), and patients that received NHT for 7–12 months (*n* = 50), as indicated. Representative images are shown. Scale bars: 100 μm.

Quantitative presentation of the data from (C). *P = 0.006 in unpaired Student's *t*-test.

A model for AR regulation of UPR in PCa cells: Liganded AR activates the IRE1α pathway and coordinately inhibits the PERK arm of the UPR. In addition, AR inhibits the proapoptotic JNK pathway that may be activated by IRE1α or other pathways. The end result of these AR effects is PCa cell proliferation and survival. An arrow with a solid line indicates direct promotion. An arrow with dashed line indicates indirect/unexplored interactions.

## Discussion

Unfolded protein response has important roles both in normal development and physiology, as well as in pathological states (Hetz *et al*, 2011). Our data show that androgens differentially affect the canonical UPR signaling arms favoring the adaptive, prosurvival pathways in PCa cells to promote growth and viability. As primary survival factors in PCa, androgens generate a UPR response favoring adaptive responses not only by activating IRE1α signaling, but also by the inhibition of the PERK-eIF2α-axis: this would prevent the maladaptive aspects of UPR from the cancer cell's perspective. Consistently, using chemical-genetic strategies, IRE1α and PERK signaling were found to have opposite effects on cell viability, where IRE1α is proliferative, whereas PERK is proapoptotic (Lin *et al*, 2009). This is also consistent with previous findings where both IRE1α and CHOP activation are directly involved in the integration of all apoptotic pathways as a result of unresolved ER stress (Tabas & Ron, 2011). To our knowledge, our study is the first to document divergent regulation of the UPR by a physiological hormone and a single transcription factor, the AR, with important pathophysiological implications.

Androgen receptor directly bound to and activated expression of the IRE1α branch at different levels thus establishing a “feed-forward” loop that potentially can regulate the output of the IRE1α arm depending on the duration/strength of the androgen signal. These data show that the IRE1α branch of the UPR is positively affected by direct actions of the liganded AR to restore homeostasis and secure cell survival (Figs6). We also show that androgens coordinately inhibit JNK signaling, resulting in proliferation and protection from apoptosis (Supplementary Fig S4C). This is consistent with previous findings on the proliferative and anti-apoptotic effects of androgens on PCa cells (Kaarbo *et al*, 2007), as well as the strong concordance between AR expression and UPR gene expression in two large cohorts of human PCa (Fig1; Supplementary Fig S1 and Supplementary Tables S1, S2 and S3). In addition, IRE1α was shown to control cyclin A1 expression and thereby promote cell proliferation in PCa cells (Thorpe & Schwarze, 2010). Furthermore, the increased expression of XBP-1S in human PCa specimens compared with normal prostate, as well as the expression profile of XBP-1S in response to hormone therapy in PCa patients (Fig7), is consistent with a role of the IRE1α arm of the UPR in PCa progression.

In direct contrast with the effects on the IRE1α arm, androgens significantly inhibited the PERK pathway. Previous studies have indicated that PERK pathway activation may be involved in either proliferation or apoptosis. One study reported that sustained PERK signaling impairs cell proliferation and promotes apoptosis (Lin *et al*, 2009), while others found PERK activation to be important for cell survival and proliferation (Urano *et al*, 2000; Bobrovnikova-Marjon *et al*, 2010). These discrepancies may be due to differences in the cell types used, or the nature and length of the activating stimuli. Our observations in PCa cells add another level of complexity in terms of PERK signaling effects on proliferation versus apoptosis: Whereas PERK activation and thus eIF2α phosphorylation is downregulated, downstream targets of this pathways, ATF4 and CHOP, were increased at the protein level upon androgen stimulation (Fig2G). This is despite the fact that *ATF4* mRNA expression is not significantly affected, whereas *CHOP* mRNA expression is decreased by androgens (Supplementary Fig S3). One possible explanation of these observations is that upon dephosphorylation of PERK and eIF2α by androgen treatment, there is a general increase in protein synthesis which compensates for the effects observed at the mRNA level. The resulting net increase in ATF4 and CHOP is significantly less than that observed with a bona fide ER stress inducer, such as TG (Armstrong *et al*, 2010; Bobrovnikova-Marjon *et al*, 2010; Chitnis *et al*, 2012). This may suggest that CHOP expression under these conditions is not high enough to trigger apoptosis in light of the strongly activated IRE1α pathway. Alternatively, since CHOP may act as a survival factor under certain conditions (reviewed in Wang & Kaufman, 2014), it may improve survival in PCa cells. Further analyses are required to uncover the details in the regulation of the PERK pathway by androgens in PCa cells.

It is clear that the regulation of IREα expression and those of XBP-1S target genes are through direct AR binding to these genes and transcriptional regulation. Although nongenomic effects, for example through crosstalk with other signaling pathways, could be in play, the fact that AR knockdown completely inhibits androgen regulation supports the view that the effects are direct. For the PERK arm, the exact mechanism of repression by androgens is not clear at present. In ChiP-Seq data sets, there is no AR binding to PERK or eIF2α genes and thus the effects could be mediated by interaction of AR with other signaling pathways (for a review, see Kaarbo *et al*, 2007). AR-mediated signaling effects could be post-transcriptional, for example at the level of translation or protein stability. Further studies are required to test these possibilities.

Previous work has shown that many cytotoxic conditions that are involved in cancer development, such as hypoxia, nutrient deprivation, and alterations in pH, trigger a set of pathways that include the ER stress response (Li *et al*, 2011). Many parts of the ER stress response protect cancer cells from death, and available data indicate that ER stress has a key role in tumorigenesis (Ma & Hendershot, 2004; Tsai & Weissman, 2010). Findings in PCa to date regarding the role of ER stress have been limited and contradictory (Segawa *et al*, 2002; Pootrakul *et al*, 2006; Daneshmand *et al*, 2007; Scriven *et al*, 2009). The data we presented here show that UPR pathways are distinctly and coordinately regulated to promote tumor survival. This opens up the possibility to selectively manipulate UPR branches as therapeutic approaches in PCa. Our findings *in vitro* and *in vivo*, including the use of a small molecule inhibitor of IRE1α to retard PCa growth, establishes a proof-of-principle for such studies in the future.

## Materials and Methods

### Cell culture

The human PCa cell line LNCaP was purchased from the American Type Culture Collection (Rockville, MD), and the human PCa cell line VCaP was a kind gift of Frank Smit (Radboud University Nijmegen Medical Centre, Nijmegen, The Netherlands). Cells were routinely kept in a humidified 5% CO_2_ and 95% air incubator at 37°C in RPMI 1640 containing 10% fetal calf serum (FCS), 50 U/ml penicillin, 50 μg/ml streptomycin and 2 mM L-glutamine (all purchased from BioWhittaker-Cambrex). The hormone responsiveness and expression of proteins characteristic to each cell line were tested on a regular basis. For the experiments, cells were plated in full medium containing 10% FCS and then preincubated in either medium containing 2% charcoal-treated (CT) FCS for 3 days or for 2 days and an additional day in medium containing 0.5% CT-FCS. For Western blot analysis, VCaP cells were cultured in 10% CT-FCS for 3 days before treatment, while for ChIP experiments, they were grown in 5% CT-FCS for 2 days before the addition of hormone. Where indicated, cells were then treated with the synthetic androgen R1881 (10^−7^ M or 10^−8^ M) or dihydrotestosterone (DHT) (100 nM) for the various time points. Both concentrations of R1881 had a similar proliferative effect on LNCaP cell growth and affected gene expression similarly. All cell lines were routinely tested and were negative of mycoplasma contamination.

### Generation of stable knockdown cells

Lentivirus-mediated stable knockdown cells were generated as described previously (Wang *et al*, 2010).

### Ectopic expression of XBP-1

LNCaP cells were transfected with the indicated siRNA (5 nM) using Lipofectamine RNAiMax reagent. One day after siRNA transfection, the cells were transfected with either vector control or Flag-XBP-1S (Addgene plasmid #21833) (Calfon *et al*, 2002). Three days after transfection, cells were harvested for Western blot analysis or split into 96-well plates before cell proliferation was assessed using the CCK-8 assay.

### RNA interference

Small interfering RNA (siRNA) was used to silence AR. The siRNA duplex used for targeting human AR was (sense strand): 5′- CUGGGAAAGUCAAGCCCAUTTdTT-3′ (Dharmacon). An siRNA targeting the luciferase gene was used as a negative control (Qiagen). 200 nM of the respective siRNAs was used. For silencing IRE1α or XBP-1, siRNA against IRE1α (Santa Cruz, sc-40705) or XBP-1 (Santa Cruz, sc-38627) or Allstar Negative Control siRNA (Qiagen) was used. siRNA was transfected into LNCaP cells using Oligofectamine or Lipofectamine RNAiMAX as per the manufacturer's instructions (Invitrogen). Where indicated, R1881 was added 1 h prior to siRNA transfection.

### Quantitative PCR

RNA extraction, cDNA synthesis, and quantitative PCR were performed as described previously (Klokk *et al*, 2007). PCR primer sequences are available upon request. A standard curve made from serial dilutions of cDNA was used to calculate the relative amount of the different cDNAs in each sample. The values were normalized to the relative amount of the internal standard GAPDH. The experiments were performed in triplicate and repeated thrice with consistent results.

### Western blot analysis

Whole-cell extracts were made as previously described (Engedal *et al*, 2002), resolved by SDS–PAGE and transferred to a PVDF membrane (Bio-Rad). The blotted membrane was blocked in 5% nonfat dry milk in Tris-buffered saline (TBS) containing 0.1% Tween (TBS–Tween) for 1 h followed by incubation with primary antibody in TBS–Tween containing 5% bovine serum albumin (BSA) for 14–16 h at 4°C. Antibodies used were against IRE1α (3294S), phospho-PERK (3179S), PERK (3192S) phospho-eIF2α (9721L), eIF2α (9722S), phospho-JNK (9251L), JNK (9252), ATF4 (11815S), cleaved caspase-3 (9661L), PCNA (13110S) (Cell Signaling), XBP-1 (sc-7160), CHOP (sc-7351), PSA (sc-7638), β-actin (sc-58670), GAPDH (sc-47724), β-tubulin (sc-9104) (Santa Cruz), α-tubulin (Sigma-Aldrich), AR (06-680) (Upstate), and phospho-IRE1α (PA1-16927) (Thermo Scientific). The membranes were then incubated with horseradish peroxidase-conjugated anti-rabbit IgG or anti-mouse IgG (Sigma-Aldrich) secondary antibodies in 5% nonfat dry milk dissolved in TBS–Tween for 1 h at room temperature. ECL Western blotting analysis system was utilized for detection of the immunoreactive bands according to the manufacturer's instructions (Amersham Pharmacia Biotech).

### Chromatin immunoprecipitation (ChIP)

ChIP experiments were carried out according to the standard protocol (Upstate Biotechnology). LNCaP or VCaP cells were plated in 15-cm tissue culture plates and cultured as described above. Cells were treated with R1881 or vehicle for 24 and 48 h followed by a crosslinking step (1% formaldehyde at 37°C), and a quenching step with 125 mM glycine. Chromatin was sonicated using the Bioruptor sonicator (Diagenode) and was immunoprecipitated with antibodies against AR (Santa Cruz, sc-816) or IgG (Vector Laboratories; I-1000). After reversal of crosslinking, immunoprecipitated DNA, as well as input DNA, was quantified by qPCR. Primers used are available upon request. Standard curves were created by 10-fold serial dilutions of an input template. The data shown are representative of at least three independent experiments.

### Xenografts

Xenografting and growth of LNCaP and VCaP cells were performed as previously described (Jin *et al*, 2013). All procedures on mice, including mouse strain, animal sex, age, number of animals allowed, and housing, were conducted according to an experimental protocol approved by the University of Oslo Institutional Animal Care and Use Committee. Briefly, five million cells were suspended in 50 μl RPMI-1640 medium and mixed with 50 μl matrigel (BD Biosciences). The mixture was then subcutaneously inoculated into male nude mice (BALB/c Nu/Nu, 5 weeks of age) in both hind flanks. Tumor size was measured weekly in two dimensions with calipers, and the tumor volume V was calculated according to the formula: V = W2 × L × 0.5, where W and L are tumor width and length, respectively. For the treatments, the tumor-bearing mice were divided into two groups where the mean tumor volumes were approximately equal. No blinding was carried out. Mice were treated by intraperitoneal injection of 0.5 mg/kg toyocamycin (Sigma) or saline solution as vehicle twice weekly until the end of the experiment.

### Immunohistochemistry

The prostate tissue microarrays (TMAs) were previously described (Klokk *et al*, 2007; Wang *et al*, 2010). After deparaffinization, antigen retrieval was done by autoclaving at 121°C for 10 min in 10 mM citrate buffer (pH 6.4). The affinity-purified XBP-1S antibody (Biolegend, #619501) was used at a dilution of 1:50 for 1 h at room temperature. The Supersensitive Detection kit (Biogenex) was used for antigen detection (Klokk *et al*, 2007). For scoring, values on a four-point scale (negative, weak, moderate and strong) were assigned to each immunostain. To compare XBP-1S expression between benign and malignant tissue, Mann–Whitney test was applied. This study was approved by the Regional Ethics Committee, REK Sør-Øst (S-07443a), and material from still living patients was included after their written consent.

For the untreated and neoadjuvant hormone therapy (NHT) samples, total of 108 prostate cancer specimens were obtained from Vancouver Prostate Centre Tissue Bank. The H&E slides were reviewed, and the desired areas were used to construct TMAs (Beecher Instruments, MD, USA). All specimens were from radical prostatectomies. Details of the material are presented in Supplementary Table S5. IHC was conducted by Ventana auto-stainer model Discover XT (Ventana Medical System, Tuscan, Arizona) with enzyme-labeled biotin streptavidin system and solvent-resistant DAB Map kit. For scoring, values on a four-point scale were assigned to each immunostain. Descriptively, 1 represents no apparent staining or very weak level of staining, 2 represents a faint or focal, questionably present stain, 3 represents a stain of convincing intensity in a minority of cells, and 4 represents a stain of convincing intensity in a majority of cells. SPSS 10.0 software was used for IHC statistical analysis. The Kruskal–Wallis test was used for analysis of correlation between XBP-1S expression and tumor grade.

### Statistical analysis

Mean and standard deviation values were calculated using Microsoft Excel software. The treatment effects in each experiment were compared using Student's two-sided *t*-test unless indicated otherwise. Values of *P* < 0.05 were considered as significant.

### Computational analysis

The expression level of AR- and UPR-associated genes (http://www.broadinstitute.org/gsea/msigdb/geneset_page.jsp?geneSetName=REACTOME_UNFOLDED_PROTEIN_RESPONSE) in PCa specimens was assessed using the gene expression datasets from two independent studies: TCGA Prostate Adenocarcinoma cohort (*n* = 190) and MSKCC Prostate Adenocarcinoma cohort (*n* = 126) (http://www.cbioportal.org/public-portal/index.do). The concordance of AR expression and UPR specific gene expression was evaluated with Pearson's correlation analysis using the software R.

## Supporting Information

Supplementary Legends

Review Process File
