# Supplementary material for: Divergent androgen regulation of unfolded protein response pathways drives prostate cancer
Source: EMBO Mol Med. 2015 Apr 11;7(6):788–801. doi: 10.15252/emmm.201404509 (PMC4459818; doi:10.15252/emmm.201404509)

Supplementary Figure 1

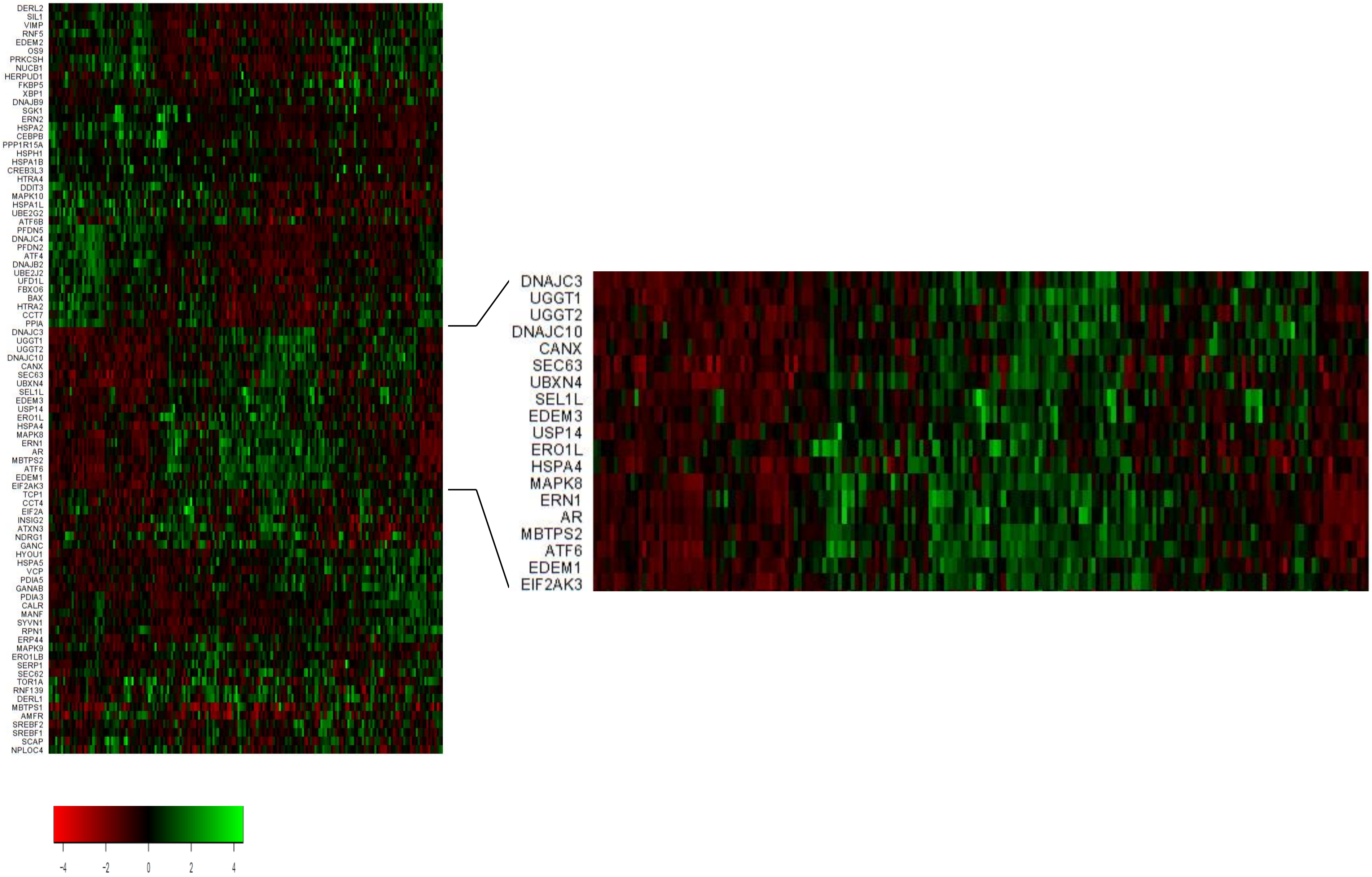

Supplementary Figure 2

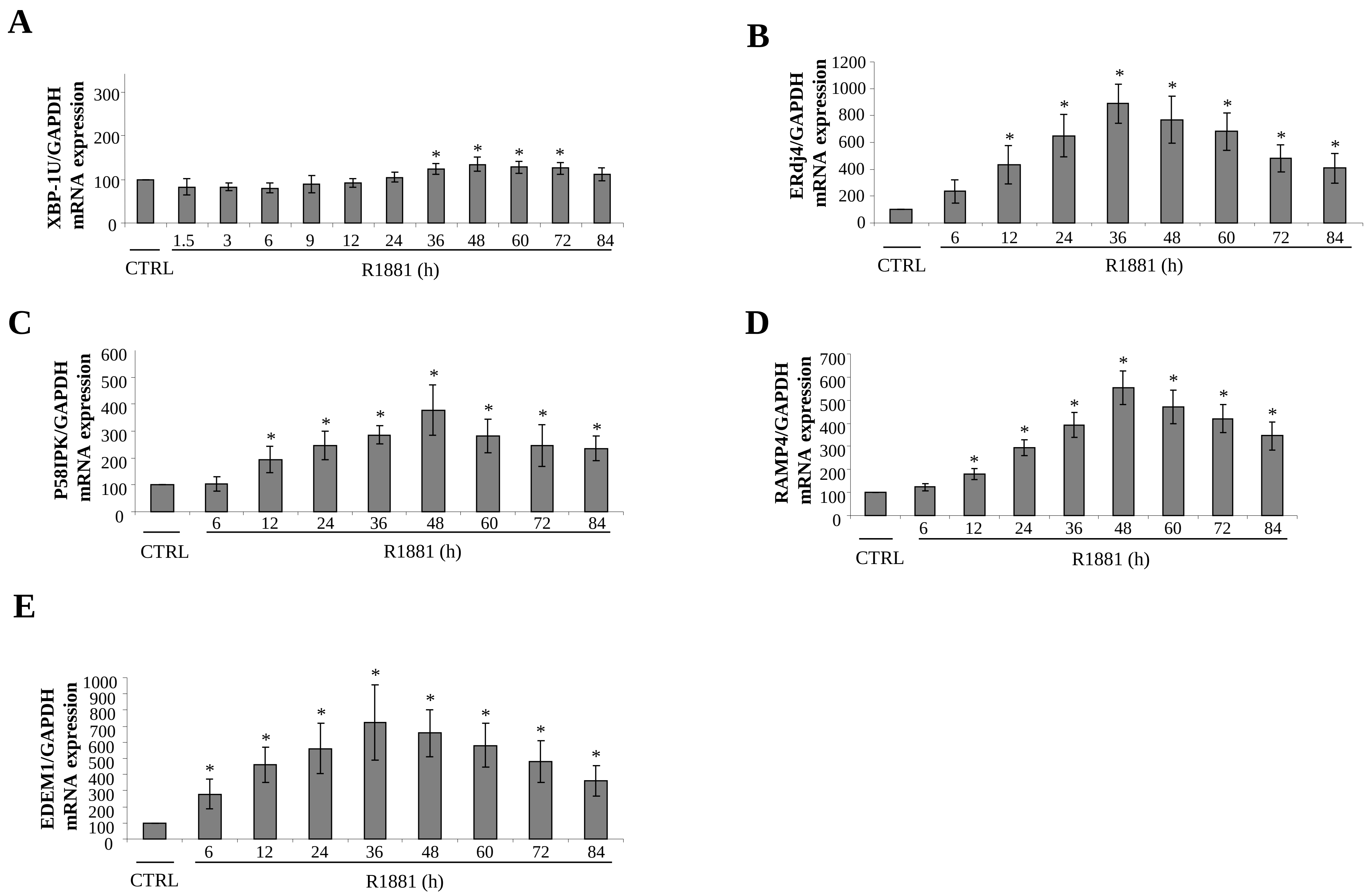

Supplementary Figure 3

A

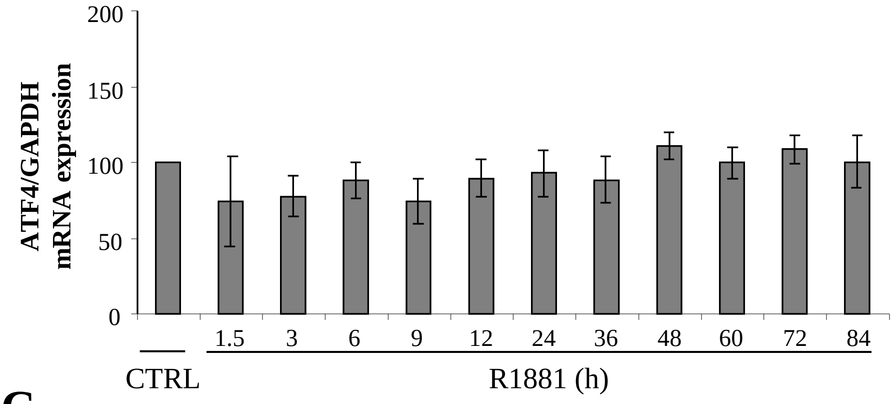

B

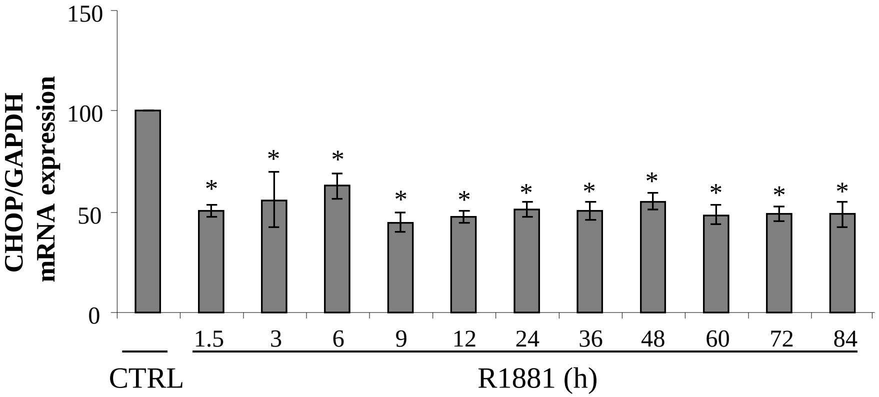

C

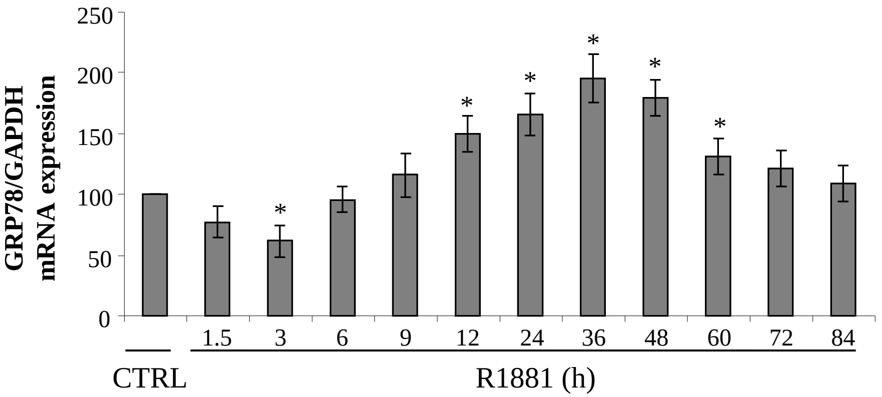

Supplementary Figure 4

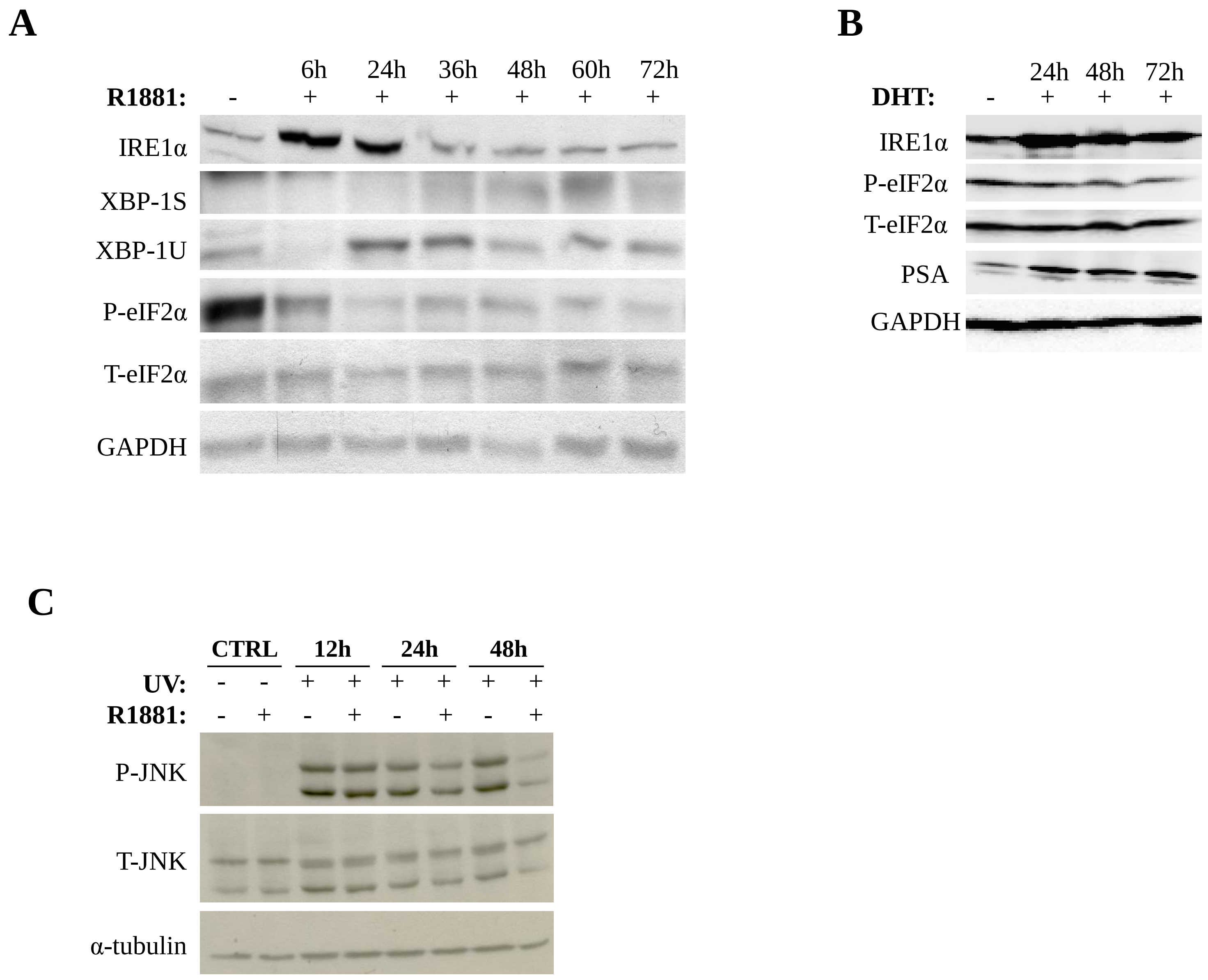

Supplementary Figure 5

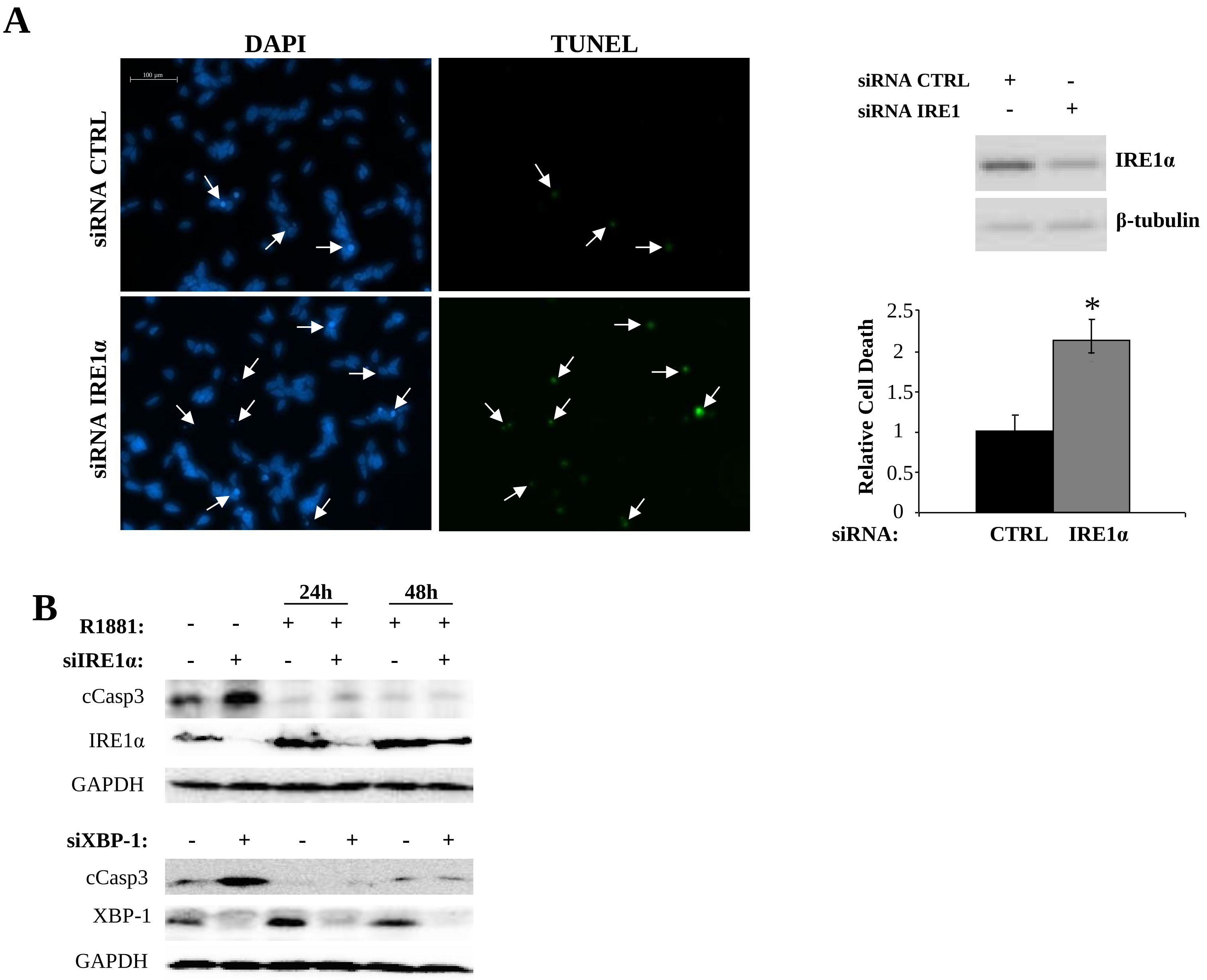

Supplementary Figure 6

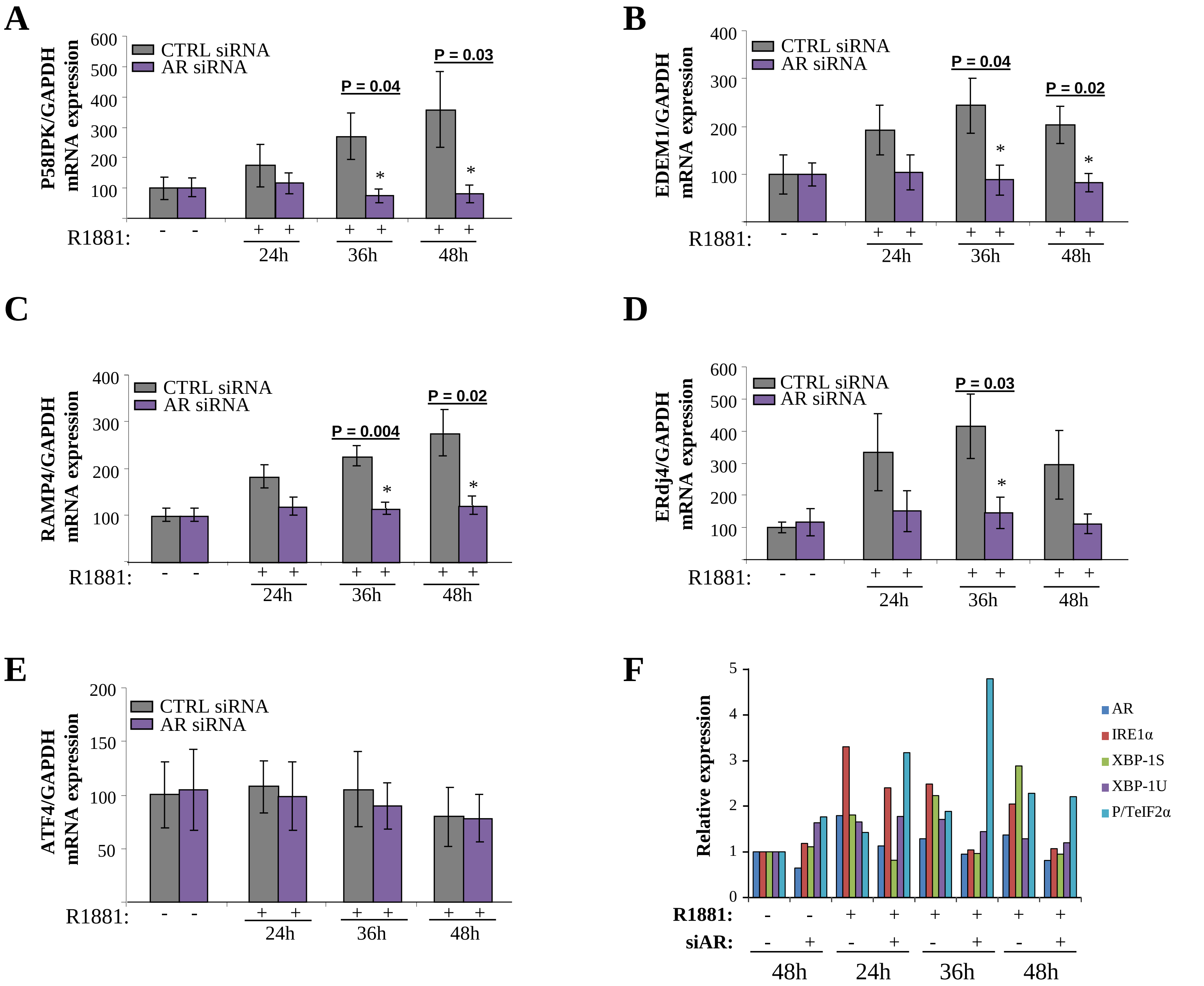

Supplementary Figure 7

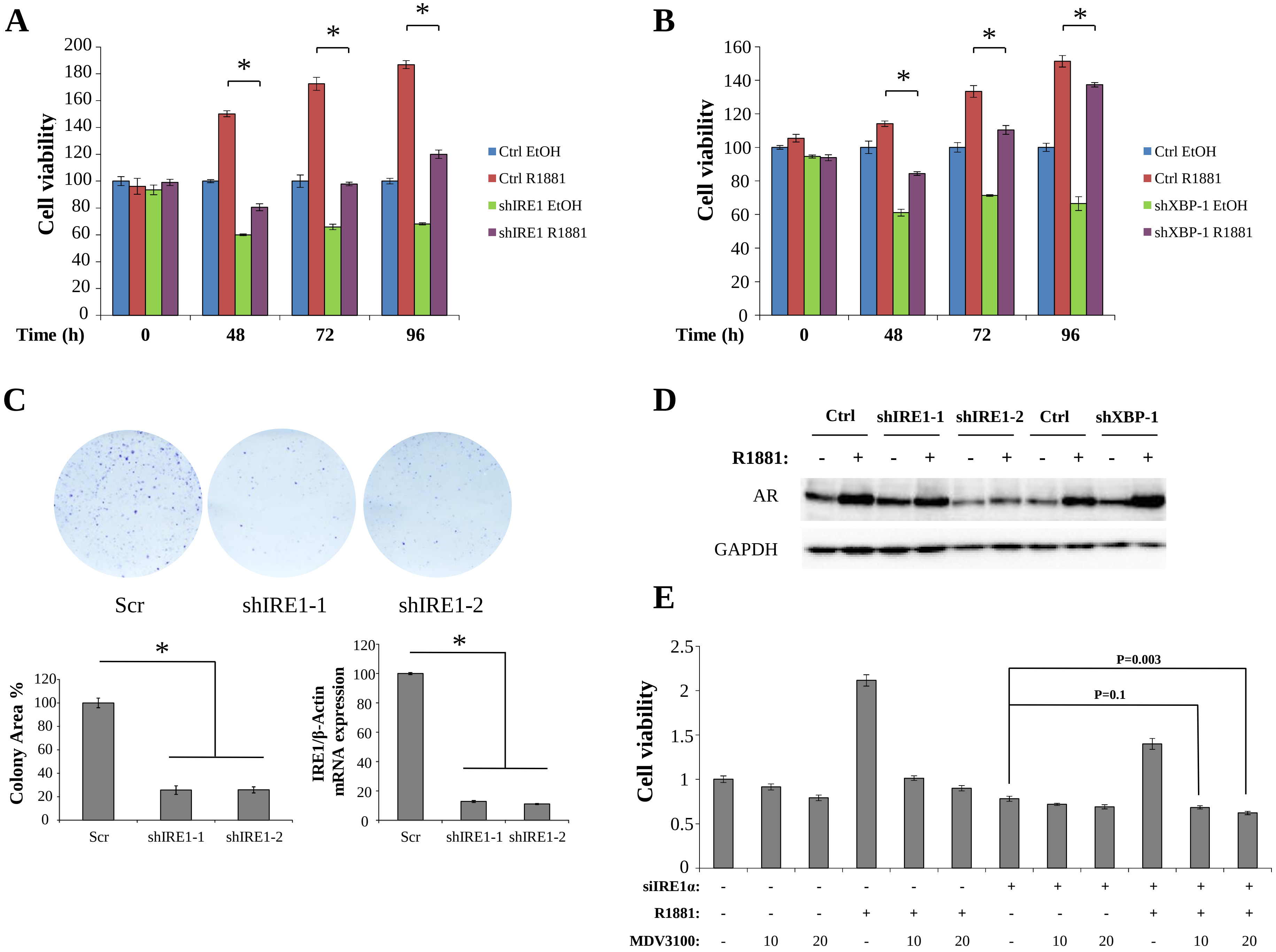

Supplementary Figure 8

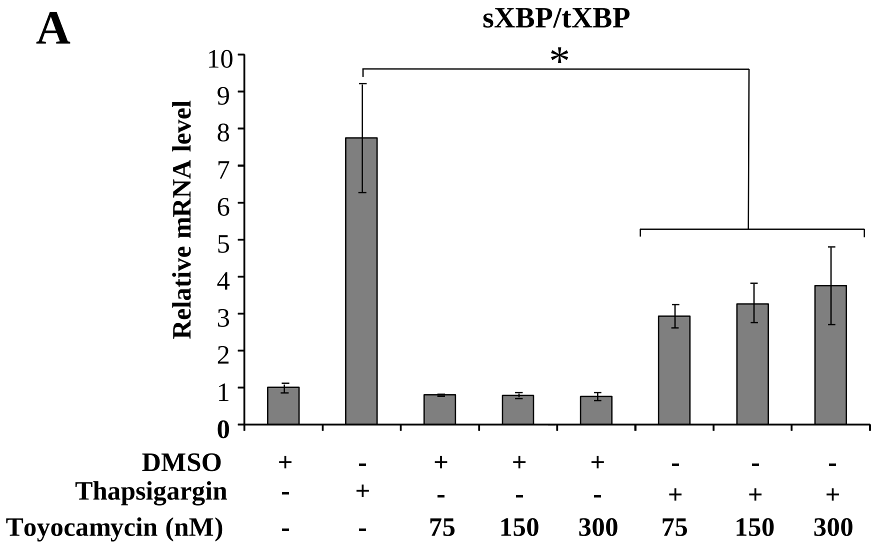

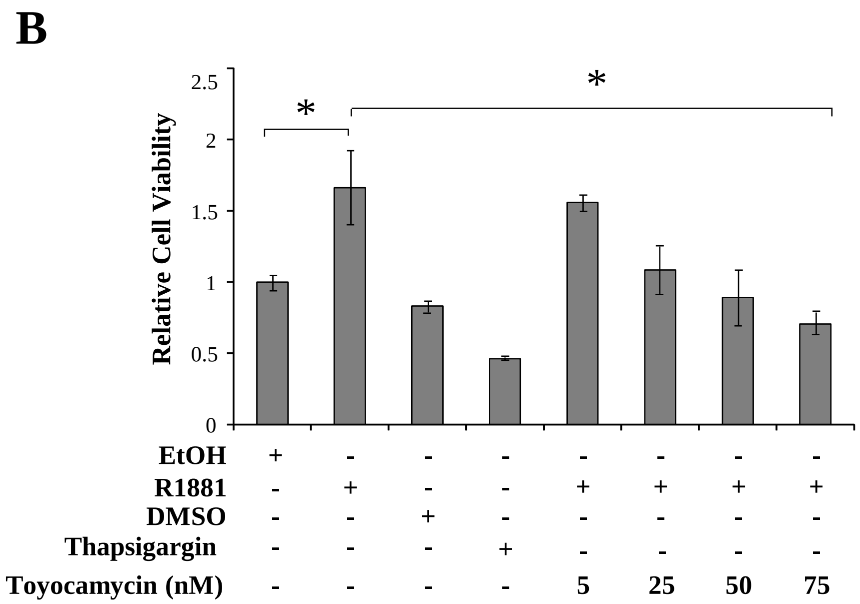

Supplement: Supplementary file 1 [file emmm0007-0788-sd1.pdf]
