# Supplementary material for: Divergent androgen regulation of unfolded protein response pathways drives prostate cancer
Source: EMBO Mol Med. 2015 Apr 11;7(6):788–801. doi: 10.15252/emmm.201404509 (PMC4459818; doi:10.15252/emmm.201404509)
Supplement: Supplementary file 3 [file emmm0007-0788-sd3.doc]

Supplementary Methods

*Cell viability assay*

LNCaP cells were plated in 2% CT-FCS containing media, pre-treated with R1881 (10^-8^ M) or vehicle control for 24 h before toyocamycin was added at the indicated concentrations. After 24 h of toyocamycin treatment, cell viability was measured using the Cell Counting Kit-8 (Sigma) according to the manufacturer’s instructions.

*TUNEL assay*

To detect apoptosis, DeadEnd^TM^ Fluorometric TUNEL system (Promega) was used according to the manufacturer’s instructions. Vectashield HardSet Mounting Medium with DAPI (Vector Laboratories) was used to mount the sections and to visualize cell nuclei. Fluorescence was observed using an Axioplan2 imaging microscope (Zeiss) at a magnification of 20x, and pictures were taken with an AxioCam MR3_2 camera (Zeiss). A minimum of 3000 cells per data point were counted, and the number of TUNEL-positive cells was expressed per 100 of the total number of cells.
