# Supplementary material for: Divergent androgen regulation of unfolded protein response pathways drives prostate cancer
Source: EMBO Mol Med. 2015 Apr 11;7(6):788–801. doi: 10.15252/emmm.201404509 (PMC4459818; doi:10.15252/emmm.201404509)
Supplement: Supplementary file 4 [file emmm0007-0788-sd4.docx]

**Supplementary Figure Legends:**

**Figure S1.** **AR expression is correlated with UPR gene expression in PCa tumors.** Assessment of possible correlation between AR and UPR associated gene expression in the global gene expression data available in the TCGA Prostate Adenocarcinoma cohort (n=190) The correlation is displayed as a heatmap generated by R program.

**Figure S2.** **Androgens regulate UPR gene expression.**

LNCaP cells were cultured and treated with R1881 for the indicated times. A) mRNA expression level of *XBP-1U*. B-E) Expression levels of XBP-1S targets *ERdj4*, *P58IPK*, *RAMP4* and *EDEM1* were determined by qPCR. Controls were treated with vehicle for 84 h and these values were set to 100. Data represent the mean of three independent experiments in triplicate. * P values ranged from 2.9x10^^-5^ to 0.04 indicating significant difference between androgen and vehicle treated cells using unpaired Student’s t-test. *P58IPK* expression in R1881 48 h compared to control P = 0.008.

**Figure S3.** **Differential regulation of UPR branches by androgens.**

LNCaP cells were cultured and treated with R1881 for the indicated times. A) mRNA expression level of *ATF4* in LNCaP cells was investigated by qPCR. B) and C) same as in A, but *CHOP* and *GRP78* mRNA levels, respectively, were assessed by qPCR. Data represent the mean of three independent experiments in triplicate. * P values ranged from 0.0003 to 0.03, indicating significant difference between androgen and vehicle treated cells using unpaired Student’s t-test. *CHOP* expression in R1881 3 h compared to control P = 0.01.

**Figure S4.** **Androgens induce the IRE1α arm and inhibit the JNK pathway.**

A) Effect of androgens on UPR arms was determined in VCaP cells which were cultured in 10% CT-FCS for three days before indicated times of treatment with R1881. B) DHT induces a UPR response in LNCaP cells. LNCaP cells were cultured in 2% CT-FCS for three days, and treated with 100 nM DHT for the indicated time points. C) JNK activation is inhibited by androgens. LNCaP cells were cultured and treated with R1881 before Western blot analysis. UV irradiation was done as described in Supplementary Methods. Data presented are representative of three independent experiments.

**Figure S5. Knockdown of IRE1α increases apoptosis of LNCaP cells.**

A) TUNEL assays were performed as described in the Supplementary Methods. Representative images (20x magnification) of LNCaP cells transfected with either IRE1α or control siRNA (50 nM) for 96 h. TUNEL positive cells appear as green fluorescent spots. Arrows point to apoptotic cells. Scale bar: 100 μm. IRE1α knockdown was confirmed by Western blot analysis and quantification of apoptosis is shown. The graph is representative of one experiment in triplicate, repeated twice with similar results. Error bars represent S.D. with *: P = 0.02 indicating significant difference between the different treatments in paired Student’s t-test. B) LNCaP cells were transfected with siRNA against IRE1α (10 µM), cultured in 2% CT-FCS medium for two days before R1881 treatment. At the indicated time points cells were harvested, proteins extracted and used in Western blot analysis.

**Figure S6. AR knockdown affects mRNA levels of XBP-1S targets, but not ATF4.**

After starvation, LNCaP cells were transfected with control (CTRL) siRNA or AR siRNA. Cells were then treated with R1881 for the indicated times. Controls were treated with vehicle for 48 h. A-E) mRNA expression levels of XBP-1S targets *P58IPK*, *EDEM1*, *RAMP4*, *ERdj4*, as well as *ATF4* were detected by qPCR. Bars represent SE. * P values are indicated showing significant difference between AR siRNA and CTRL siRNA transfected cells in unpaired Student’s t-test. F) Quantification of the Western blot in Figure 3F. Protein levels were quantified using ImageJ.

**Figure S7. IRE1α depletion leads to growth retardation and a decrease in cell viability.**

A) Equal amounts of LNCaP Ctrl and shIRE1 cells were plated in 96-well plates, cultured in 2% CT-FCS for two days and then treated with R1881. Cell viability was assessed at each indicated time point. The graph is representative of one experiment with six replicates for each group, repeated twice with similar results. Error bars represent SEM. P-values comparing control R1881 treated cells to shIRE1 R1881 treated cells for 48, 72 and 96 h were P = 1.82x10^^-9^, P = 3.38x10^^-8^, P = 2.38x10^^-8^ indicating significant difference using paired Student’s t-test. B) LNCaP Ctrl and shXBP-1 cells were treated as in A. The graph is representative of one experiment in six replicates. Error bars represent SEM. P-values comparing control R1881 treated cells to shXBP-1 R1881 treated cells for 48, 72 and 96 h were P = 3.81x10^^-8^, P = 0.0003, P = 0.01 indicating significant difference using paired Student’s t-test. C) Control LN-Scr (Scr), LN-shIRE1-1 (shIRE1-1), LN-shIRE1-2 (shIRE1-2) cells were cultured for three weeks. The colonies formed were stained with crystal violet and photographed. The extent of IRE1α knockdown was determined by Western blot analysis. The area covered by colonies was quantified using the Gene Tools software (SynGene). The data are representative of three experiments in triplicate. Error bars represent SEM. Paired Student’s t-test was used to evaluate significant difference. P values comparing area covered by colonies between Scr to shIRE1 and shXBP-1 were P = 4.38x10^^-7^ and P = 1.81x10^^-7^, respectively. P values comparing IRE1α mRNA expression between Scr to shIRE1 and shXBP-1 were P = 0.0001 and P =7.68x10^^-5^. D) AR levels in Ctrl, shIRE1 and shXBP-1 cell lines. Cells were harvested, protein extracted and subjected to Western blot analysis. E) LNCaP cells were transfected with 10 nM siIRE1α or control siRNA, cultured in 2% CT-FCS for three days, and treated with 1 nM R1881 and/or MDV3100 for the indicated concentrations. The CCK-8 assay was used to measure cell viability after two days of treatment. The graph represents one experiment, repeated three times with similar results. Error bars represent SEM. * P values using paired Student’s t-test are indicated.

**Figure S8. Inhibition of IRE1α using a small molecule inhibitor leads to decreased cell viability.**

A) Inhibition of XBP-1 splicing in response to toyocamycin was assessed by qPCR. LNCaP cells were pre-treated with thapsigargin (100 nM) prior to treatment with the indicated concentrations of toyocamycin for 12 h. Error bars indicate SD. P values comparing thapsigargin alone to toyocamycin 75, 150 and 300 nM were P = 0.009, P = 0.01 and P = 0.01, respectively, using paired Student’s t-test. B) LNCaP cells were pre-treated with R1881 for 24 h before treatment with the indicated concentrations of toyocamycin. Cell viability was measured after 24 h using the CCK-8 assay. The data represent one experiment done in triplicate. Error bars indicate SD. Paired Student’s t-test was used to evaluate significance, P = 0.02 comparing ethanol to R1881, P = 0.03 comparing R1881 alone to R1881 and toyocamycin 75 nM.

**Tables**

Table S1. Correlation of AR and UPR gene expression in the TCGA prostate cancer cohort.

Table S2. P values of Figure 1 (Differential expression of UPR genes in AR high, AR medium and AR low samples from TCGA provisional prostate cancer cohort).

Table S3. Correlation of AR and UPR gene expression in the MSKCC prostate cancer cohort.

Table S4. XBP-1S staining by IHC in tissue microarrays ([Wang et al, 2010](#_ENREF_37)).

Table S5. Clinical parameters of samples used in the tissue microarray to stain for XBP-1S. Samples were from radical prostatectomy patients that had received either no treatment or Neoadjuvant Hormone Therapy (NHT) for 1-6 or 7-12 months. Gleason score, PSA and testosterone as well as clinical stage is given, in addition to other parameters.
