# Supplementary material for: Divergent androgen regulation of unfolded protein response pathways drives prostate cancer
Source: EMBO Mol Med. 2015 Apr 11;7(6):788–801. doi: 10.15252/emmm.201404509 (PMC4459818; doi:10.15252/emmm.201404509)
Supplement: Supplementary file 5 [file emmm0007-0788-sd5.pdf]

EMBO Molecular Medicine

Divergent androgen regulation of unfolded protein response pathways drives prostate cancer

Xia Sheng, Yke Jildouw Arnoldussen, Margrethe Storm, Martina Tesikova, Hatice Zeynep Nenseth, Sen Zhao, Ladan Fazli, Paul Rennie, Bjørn Risberg, Håkon Wæhre, Håvard Danielsen, Ian G. Mills, Yang Jin, Gökhan Hotamisligil and Fahri Saatcioglu

Corresponding author: Fahri Saatcioglu, University of Oslo

Review timeline:

Submission date:	24 July 2014
Editorial Decision:	28 August 2014
Revision received:	06 February 2015
Editorial Decision:	26 February 2015
Revision received:	02 March 2015
Accepted:	09 March 2015

Transaction Report:

(Note: With the exception of the correction of typographical or spelling errors that could be a source of ambiguity, letters and reports are not edited. The original formatting of letters and referee reports may not be reflected in this compilation.)

Editor: Roberto Buccione

1st Editorial Decision 28 August 2014

Thank you for the submission of your manuscript to EMBO Molecular Medicine. We have now heard back from the three Reviewers whom we asked to evaluate your manuscript.

We are sorry that it has taken longer than usual but this was to some extent to be expected given the vacation period.

You will see that while two Reviewers are more supportive of your work, albeit expressing numerous concerns, one is quite negative. All considered, significant issues are raised that prevent us from considering publication at this time. I will not dwell into much detail as their comments are detailed. I would like, however, to highlight a few main points.

Reviewer 1 would like to see more convincing experimental support for the claims made. For instance s/he notes that conclusive evidence that androgens activate the IRE1alpha UPR branch is lacking and that positive controls in UPR signalling evaluation would be required. The Reviewer also feels that a bona fide ER stressor should be used to test the effect of androgens on JNK. Finally, Reviewer 1 would also like to better understand the clinical relevance of your work and wonders about the effects of toyocamicin in an androgen deprived setting.

Reviewer 3 is also globally positive but also raises specific concerns that require your action. I would like to mention his/her request for more detailed information on the prostate samples, especially since recurring samples are also used. Importantly, and connected to this, and similarly to Reviewer 1 s/he also raises the issue of the (clinically very relevant) castration-resistant stage of PCa and the possible role of UPR in that setting.

Reviewer 2 is clearly less positive but, ultimately, raises much the same issues as the other two, namely concerning mechanistic conclusiveness, detailed description of the clinical samples, and the androgen independent setting.

I will not be requiring you to perform a full array of experiments in the androgen resistant setting experiments (nor to test other androgens) (provided all other issues are carefully and fully dealt with). I do, however encourage you to develop your study as far as realistically possible in this sense and in any case carefully and extensively discuss the issue of the transition to the castration-resistant stage for your next, revised version to strengthen your findings and increase their impact.

Considered all the above, while publication of the paper cannot be considered at this stage, we would be prepared to consider a substantially revised submission, with the understanding that the Reviewers' concerns must be addressed as outlined above with additional experimental data where appropriate and that acceptance of the manuscript will entail a second round of review.

I look forward to seeing a revised form of your manuscript as soon as possible.

***** Reviewer's comments *****

Referee #1 (Remarks):

Remarks to the authors:
The manuscript by Arnoldussen and colleagues provide evidence for a possible crosstalk between UPR signaling and androgen through the androgen receptor (AR) in in vitro and in in vivo studies with prostate cancer models. Androgen seems to induce IRE1α levels and activate IRE1α signaling, in contrast to the PERK pathways that is inhibited. This result correlates with findings from human samples where levels of XBP1s are increased in patients with prostate cancer. In addition, the authors also demonstrate that toyocamycin decrease tumor formation in xenografts of prostate cancer.
General comments:
Overall the results and data are convincing and well-presented and deserved publication in this journal. However some controls and experiments are required to improve the in vitro studies that are in some aspects weak.

Specific Comments:
There is increasing evidence suggesting that the UPR is suppressed according to the stage of prostate cancer. Base on this, figure 1 describes a correlation between AR expression and UPR markers. Is there any relation of this stratification with the stages of the tumors?
The authors mentioned that "androgens activate the IRE1α branch of the UPR"; however, more experiments are needed to prove this idea. Based that androgens also increase the levels of IRE1α, it is necessary to measure and compare the kinetics of activation of IRE1α (phosphorylation, oligomerization, XBP1 mRNA splicing) in respect to the increase on the levels of IRE1α. In addition, other parameters of IRE1α signaling could be evaluated such as RIDD and JNK activation.
In the same point, figure 2 and S2 describe a significant increase of XBP-1s mRNA after 12 hrs that correlate with the expression of transcriptional targets such as Erdj4, EDEM, etc; however protein levels of XBP1s appears at 48 hrs of treatment with androgens. Can the authors explain this observation? It is possible to have XBP-1 independent effect, maybe mediated by ATF6 since they have shared target genes?
It would be useful to add a positive control with tunicamycin or thapsigargin in all experiments evaluating UPR signaling.
The authors also suggest that PERK signaling might be decreased by androgens and ATF6 is partially increased; however there are no proper experiments to address this point. Androgens decrease eIF2a phosphorylation; however there is no stimulus to activate the PERK branch. Does androgen affects PERK levels or the phosphorylation status or ATF4 protein levels? Can the androgens suppress the activation of PERK under ER stress conditions? The authors also mentioned that ATF4 mRNA levels are intact, but there is a decrease of CHOP expression; however the changes observed are really low. Can you comment these observations?

In addition, the authors also suggest that JNK activation is decreased by androgens; however this is done with UV exposure. This should be done with a classical ER stressor, to evaluate the activation of JNK mediated induced by IRE1a.
Figure 3 demonstrate the role of AR using siRNA in the increase of IRE1α and XBP-1s under androgen treatments. As mentioned before, it would be useful to add a control with tunicamycin or thapsigargin to see if AR have a role in the UPR.
Figure 5 described that silencing of IRE1α and XBP-1 decrease cell growth in vitro and in vivo. This is also validated using an IRE1α inhibitor such as toyocamycin (Figure 6). In vitro studies using toyocamycin demonstrate that toyocamycin rescue the proliferation effects mediated by androgens in LnCap cells in a dose dependent manner. Can androgens rescue the defects of growth and apoptosis observed silencing IRE1α and XBP-1.
On the other side, for prostate cancer therapy it is commonly used an androgen deprivation therapy. So, what happened if in in vivo experiments using toyocamycin are done in absence of androgens. Can these two therapies synergize and decrease even more the tumor formation?

Minor comments:
Update references related with UPR information
Improve and quantified images of western blot of figure 3F
Describe the methods used for measure growth in figure 5C and D. Why scramble controls are different?

Referee #2 (Remarks):

General comments:

Arnoldussen and colleagues presented their data on the impact of androgen receptor mediated effects on the unfolded protein response (UPR) pathways, proposing that these are important signaling events as pro-survival mechanisms. This is an important topic. Importantly, using stable gene silencing and small molecule inhibitor were employed to target a sub-pathway downstream of the UPR implicated in this report. However and unfortunately, the significance of the observed effects is highly unclear in terms of the specific relevance to androgen receptor related function. In addition, the data on clinical correlates were considered preliminary and descriptive. The report suffers from the lack of insight into the molecular basis of selective UPR signaling mediated by AR, and whether this is a generic phenomenon or limited to specific cell lines.

Overall, there were a number of fundamental issues as well as the need to proof reading to correct a number of grammatical and formating issues:
Page 4, line 5 - bracket missing
Page 6 line 12 - what is the e.g.? The reference can be cited without 'e.g.'

Specific comments:
Introduction
The cell fate upon UPR activation is described well. However, other cellular pathways including autophagy or mitophagy may be relevant to be included. The involvement of XBP1 splicing was mentioned on several occasions. It would be useful for the reader to have further information on how androgen receptor would affects this splicing event.

Results
Figure 1
There are several points not clear in terms of methodology in this data mining exercise.
• How were the UPR genes selected? It would be worthwhile correlating the UPR gene signature with both hormone niave and castrate resistant prostate cancers rather than AR levels per se.
• AR levels were grouped into three groups. The intermediate group does not seem to be informative. What was the data if AR expression was simply considered in two groups? For panel B, for readers' ease, it would be useful if the individual p values for each of the genes highlighted to be included.

• VCaP data - should at least be shown in supplementary rather than simply "data not shown"
Other clinical parameters should be included in the analysis, including tumour stage and grade.
Given the expression of AR in stroma, it would be useful to have information on the relative amount of stroma included in the samples.
Supplementary Figure 1 - it is not clear what the gene list represents? What were the criteria used to generate the gene list?

Figure 2
It should be made clearer that data in panels A-C were from in vitro studies while that of D-F were from xenografts.
In the vitro work, besides R1881, was there additional confirmation data using another androgen such as DHT?
Also, what is the response of the CWR22 to R1881? Is the converse true?
JNK data is interesting, but is JNK signaling still inhibited in the IRE1 knockdown?
Supplementary Figure 2 - Panel F - In the control lanes, it would appear that total JNK level was increased following R1881 treatment, despite undetectable p-JNK. There was no mention of this.

Figure 3
"....CHOP is directly downregulated by AR' - This reviewer is unsure if the data presented here would necessarily confirm the 'direct' nature of the regulation. The statement would suggest that CHOP is transcriptional target of AR.
Panel F - Comparing to data shown in Figure 2C, the expression of XBP-1S, p-eIF2a and total-eIF2a (control lanes) seemed not to be consistent between the two figures.
Supplementary Figure 3B - CHOP level seemed to be suppressed for a long period (up to 84 hours) without any evidence of recovery. Is this not surprising?
It would be good to show the data for VCaP.

Figure 4
Are the ChIP data reversed by the presence of anti-andogens?
What about AR binding to XBP-1S? Is there any data on this from the ChIP?

Figure 5
It is not clear how the manipulation of IRE1 and XBP1 interact with the AR status, and vice versa.
Also, the use of anti-androgen would be useful here.
The data (both in vitro and in vivo) would be strengthened by inclusion of more than one derived clone for each KD experiment.
Panels E and F are effectively the same data. Panel F may be considered to be in the supplementary information. It is unclear what is the relevance to AR mediated growth in this experiment. It is highly likely that such growth suppression would be observed in AR negative cells.
Is apoptosis the sole reason for difference in tumour size? Any difference in proliferation rates, perhaps assessed by Ki67 staining?
Supplementary Figure 5 - Is the observed increase in apoptosis driven by an increase in JNK signaling? Panel D - TG should be given in full

Figure 6
The use of toyocamycin is interesting. Looking at the literature, it appears to be an inhibitor of rRNA processing as well as Rio 1 (Kiburu et al, Plos One 2012). It would be important to have reassurance that the observed effects were related to IRE1a. Of course, the relationship to AR function would still need to be addressed.
Like Figure 5, Panel B and D duplicated data in A and C, and could be included as supplementary information.
What about the effects of castration?

Figure 7
Overall, data seem to be preliminary and clinical relevance of the magnitude of the change remains unclear.
Panel B - It is unclear what the p value refers to.
Panel C - It is difficult to interpret without more information on tumour grade and stage; respective PSA levels would also be good. The response may be variable within the neo-adjuvant group.

Panel D - not sure what the p value refers to. Also, is the XBP-1S level significantly different in the recurrent group?
How does XBP-1S levels correlate with AR in human disease?

Other points:
What is the role for IRE1 and XBP1 loss in CaP cells that do not have AR? Is this response still present?

Referee #3 (Remarks):

The unfolded protein response (UPR) is an in-built homeostatic mechanism to maintain endoplasmic reticulum (ER) function. UPR signaling is activated by various conditions, such as differentiation of secretory cells, as well as in disease states. Prostate cancer (PCa) originates in a highly secretory organ that is regulated by hormonal signals, in particular androgen signaling. It is thus possible that PCa cells may have developed ways to engage the ER adaptive responses and hormonally regulate UPR to support tumorigenesis. The authors show that the canonical UPR pathways are directly and divergently regulated by androgens in PCa cells, which are critical for PCa survival. Androgen receptor (AR) bound to gene regulatory sites and activated the IRE1α branch, but simultaneously inhibited PERK signaling. IRE1α knockdown significantly increased apoptosis of PCa cells in vitro and its expression was decreased in human xenografts upon androgen withdrawal and regression. Moreover, IRE1α inhibition or its target XBP1, profoundly inhibited PCa cell growth in vitro as well as tumor formation in preclinical models of PCa in vivo. Consistently, AR and UPR gene expression were correlated in human PCa wherein spliced XBP1 expression was significantly upregulated compared with normal prostate. These data establish a genetic switch orchestrated by a single transcription factor, the AR that divergently regulate the canonical UPR pathways and suggest that targeting of IRE1α signaling may have utility as a novel therapeutic approach in PCa.

The paper is interesting and provides novel results in establishing a role for UPR in prostate cancer. However, there are some issues that need to be address/clarified to improve the manuscript for publication:

1) Abstract. "Prostate cancer (PCa) originates from a highly secretory cell type that is regulated by hormonal signals". This sentence of the Abstract is confusing. Does it refer to the prostate as secretory organ? Please clarify here and on page 4.
2) Page 4: "PCa originates from a highly secretory cell type that is regulated by hormonal signals, in particular androgen signaling, via the androgen receptor (AR), which is important in all phases of PCa" It would be important to mention that androgen may have a less prominent role in castration-resistant stage of prostate cancer.
3) Page 5: "in VCaP cells, an independent androgen responsive PCa line" I would change independent with another.
4) Figure 1A: Please enlarge the fonts of gene names. They are very difficult to read.
5) The description of the results presented in Figure 2 jumps from Panel A-B to D and back to see. It is not easy to follow.
6) Figure 5: Panel C and D. A colony formation assay is not exactly measuring proliferation rate of cells but foci formation in monolayer. In addition these experiments are performed in serum and therefore they do not really prove that IRE1 and XBP1 are required for androgen-dependent cell growth of LNCaP cells. It is important to show results of growth curve experiments after several time points of androgen stimulation.
7) Supplementary Figure 5D: What is TG? This assay measures cell viability not cell growth. Please correct the Y-axis to "Relative Cell Survival".
8) Page 9 and Figure 7A. "XBP-1S protein was weakly expressed in the benign prostate, predominantly in epithelial cells, and its expression was significantly increased in PCa specimens compared to normal cells" XBP1S expression in benign prostate is not exactly weak but it is definitely enhanced in tumor tissue. I would change to "XBP-1S protein was expressed in the benign prostate, predominantly in epithelial cells, and its expression was significantly increased in PCa specimens compared to normal tissue controls".

9) Page 9: "In addition, XBP-1S expression was reduced following neoadjuvant hormone therapy, remained low in patients that responded to therapy, and increased upon PSA recurrence indicating that XBP-1S is regulated by AR in vivo and may contribute to castrate resistant disease (Figure 7C-D). These data show that the activity of the IRE1α arm of the UPR is deregulated in human PCa and may have a role in disease progression".
The details of the various prostate tissues are mentioned as reported in Table S1, which is instead a list of genes. Please include the specimen characteristics, which are important for the interpretation of the data presented in figure 7C and D.
Are "recurrent" samples castration-resistant and insensitive to hormone-withdrawal therapy? If so, it would be important to better discuss the possible role of UPR genes in castration-resistant stage of prostate cancer. Is XBP staining in recurrent samples statistically different from 1-6 and 7-12 samples?
How the authors envision a possible role of IRE in castration-resistant stage when the manuscript data support an androgen-dependent role for UPR proteins?

1st Revision - authors' response 06 February 2015

Reviewer 1

1. *There is increasing evidence suggesting that the UPR is suppressed according to the stage of prostate cancer. Based on this, figure 1 describes a correlation between AR expression and UPR markers. Is there any relation of this stratification with the stages of the tumors?*

   - We have performed GSEA analysis with the UPR gene set in two prostate cancer cohorts (GSE6919 and GSE35988). Although there is a trend for a decrease in UPR gene expression in metastatic tumors compared to localized tumors, the difference did not reach significance. These data suggest that there is no clear relationship between UPR gene expression and tumor stage.

2. *The authors mentioned that androgens activate the IRE1 branch of the UPR; however, more experiments are needed to prove this idea. Based that androgens also increase the levels of IRE1, it is necessary to measure and compare the kinetics of activation of IRE1 (phosphorylation, oligomerization, XBP1 mRNA splicing) in respect to the increase on the levels of IRE1. In addition, other parameters of IRE1 signaling could be evaluated such as RIDD and JNK activation.*

   - We have now conducted additional experiments to address the points raised by the Reviewer. We found that androgen treatment induces IRE1α phosphorylation in addition to increasing total IRE1α protein levels (Fig. 2F). The time course of XBP-1 splicing has been presented in Fig. 2B and correlates very closely with the increase in IRE1α expression and activation (Fig. 2A, 2F). As for JNK activation, we have previously shown that JNK activity induced by a common ER stress inducer, thapsigargin, is efficiently inhibited by androgens in prostate cancer cells (Lorenzo and Saatcioglu, 2008). We also show an example of this in the present manuscript where UV-induced JNK activation is efficiently inhibited by androgens (Figure E4C). These data strongly support the view that IRE1α activation by androgens induces the survival effects of IRE1α through splicing of XBP-1, whereas the pro-apopotic effects are inhibited. This is further illustrated by the fact that Caspase 3 activation is significantly induced upon IRE1α knockdown (Figure E5B).

3. *In the same point, figure 2 and S2 describe a significant increase of XBP-1s mRNA after 12 hrs that correlate with the expression of transcriptional targets such as Erdj4, EDEM, etc; however protein levels of XBP1s appears at 48 hrs of treatment with androgens. Can the authors explain this observation? It is possible to have XBP-1 independent effect, maybe mediated by ATF6 since they have shared target genes?*

- The peak expression of XBP-1S targets is at 36-48 h whereas XBP-1S expression slowly increases during the time of induction reaching significant levels by 48h and continuing to increase. There are two possibilities in explaining these data, where the target expression is slightly earlier than the maximal XBP-1S expression. First, as we show herein, the XBP-1S targets are directly affected by the androgen receptor (AR) (Figure E2); thus, even before XBP-1S is available, some expression of the XBP-1S targets may occur due to activation by AR. Second, lower than maximal levels of XBP-1S may be sufficient to induce expression of its targets. In addition, as the Reviewer suggests, this slight discrepancy could be due to other pathways that may affect XBP-1S target gene expression one of which is ATF6α. Given that there is very modest increase in ATF6α target GRP78 upon androgen stimulation (Figure E3C), we feel that this is unlikely.

4. *It would be useful to add a positive control with tunicamycin or thapsigargin in all experiments evaluating UPR signaling.*

   - In all initial Western blots assessing UPR markers, a thapsigargin treated sample was run alongside other samples to verify the band size and the cells' responsiveness to UPR. Shown below is a representative Western blot of thapsigargin treated LNCaP cells showing different UPR markers [Figure omitted upon Authors' request].

5. *The authors also suggest that PERK signaling might be decreased by androgens and ATF6 is partially increased; however there are no proper experiments to address this point. Androgens decrease eIF2a phosphorylation; however there is no stimulus to activate the PERK branch. Does androgen affects PERK levels or the phosphorylation status or ATF4 protein levels? Can the androgens suppress the activation of PERK under ER stress conditions? The authors also mentioned that ATF4 mRNA levels are intact, but there is a decrease of CHOP expression; however the changes observed are really low. Can you comment these observations?*

   - New data that we provide in Figure 2G shows that both phospho-PERK and total PERK levels are markedly decreased upon androgen treatment in LNCaP cells. This is consistent with our previously presented data showing significantly reduced levels of phosphorylated eIF2α upon androgen treatment. We also provide new data showing that, surprisingly, ATF4 and CHOP protein levels are modestly increased in a time dependent manner upon androgen treatment (Figure 2G). The increase in ATF4 and CHOP, despite PERK-eIF2α downregulation, and a decrease in *CHOP* mRNA and no significant change in *ATF4* mRNA levels, could be a result of an increase in general protein synthesis as a consequence of androgen-mediated PERK and eIF2α dephosphorylation. This is now reviewed in the Discussion.

   - As suggested by the Reviewer, we have also determined whether androgens can suppress PERK activation under ER stress conditions. As shown in Rebuttal Figure 2 [Figure omitted upon Authors' request], androgens were unable to suppress p-eIF2α levels induced by a strong ER stressor, such as thapsigargin. Furthermore, there may even be additive effects on the IRE1α pathway. Further work is required, such as testing additional ER stressors, but these data suggest that under ER stress conditions the androgen effects may be divergent for the different arms of UPR.

6. *In addition, the authors also suggest that JNK activation is decreased by androgens; however this is done with UV exposure. This should be done with a classical ER stressor, to evaluate the activation of JNK mediated induced by IRE1a.*

   - Please see the response to point 2 above.

7. *Figure 3 demonstrates the role of AR using siRNA in the increase of IRE1 and XBP-1S under androgen treatments. As mentioned before, it would be useful to add a control with tunicamycin or thapsigargin to see if AR have a role in the UPR.*

   - Please see the response to point 5.

8. *Figure 5 described that silencing of IRE1 and XBP-1 decrease cell growth in vitro and in vivo. This is also validated using an IRE1 inhibitor such as toyocamycin (Figure 6). In vitro studies using toyocamycin demonstrate that toyocamycin rescue the proliferation effects mediated by androgens in LnCap cells in a dose dependent manner. Can androgens rescue the defects of growth and apoptosis observed silencing IRE1 and XBP-1?*

   - We have now performed the experiments suggested by the Reviewer. As shown in Figure E7A and B, androgen treatment rescued, at least in part, the decrease in proliferation observed in both shIRE1 and shXBP-1 (knockdown) cell lines. The rescue was almost complete in the shXBP-1 cells compared to approximately 60% in shIRE1 cells. This is interesting and suggests that effects of AR signaling on IRE1α and XBP-1, although they are in the same signaling pathway, may be divergent. Consistent with this picture, Caspase 3 cleavage that is induced upon IRE1α and XBP-1 knockdown is reversed upon androgen treatment (Figure E5B). These data suggest that androgens can rescue, at least in part, both anti-proliferative and apoptotic responses upon silencing of the IRE1α pathway.

9. *On the other side, for prostate cancer therapy it is commonly used an androgen deprivation therapy. So, what happened if in in vivo experiments using toyocamycin are done in absence of androgens. Can these two therapies synergize and decrease even more the tumor formation?*

   - We have now done *in vitro* cell viability experiments using toyocamycin in combination with the anti-androgen MDV3100. As shown in Rebuttal Figure 3 [Figure omitted upon Authors' request], toyocamycin does not synergize with AR pathway inhibition for effects on cell viability (e.g. compare R1881 alone, Toyocamycin alone (25 nM), MDV3100 alone (10 μM), R1881+Toyocamycin (25 nM), R1881 + MDV3100 (10 μM), and R1881+ Toyocamycin (25 nM) + MDV3100 (10 μM)).

Minor comments:
Update references related with UPR information

- We have now updated the references related to UPR.

Improve and quantified images of western blot of figure 3F

- The quantification of the Western blot in Figure 3F is now presented in Figure E6F.

Describe the methods used for measure growth in figure 5C and D. Why scramble controls are different?

- Colony formation assay was used on cell lines stably expressing either shRNA against XBP-1, IRE1α or scrambled control. The two control cell lines shown were generated independently at different times and therefore there is a modest difference in their proliferation rate.

Reviewer 2
Page 4, line 5 - bracket missing
This has now been corrected.

Page 6 line 12 - what is the e.g.? The reference can be cited without 'e.g.'
Since this is only a representative reference, we inserted e.g., meaning 'for example'.
Additional comments:

1. *The cell fate upon UPR activation is described well. However, other cellular pathways including autophagy or mitophagy may be relevant to be included. The involvement of XBP1 splicing was mentioned on several occasions. It would be useful for the reader to have further information on how androgen receptor would affects this splicing event.*

   - Both autophagy and mitophagy are interesting focus areas with a clear association to ER stress, but we feel that they are beyond the scope of this paper. However, we have included references to these important processes and their connection to UPR in the Introduction. Regarding the possible mechanism of XBP-1 splicing, our data show that IRE1α and XBP-1 expression are directly upregulated by AR at the level of transcription. When IRE1α levels go up, splicing of XBP-1 will be induced which will increase XBP-1S levels. The close correlation in the time course of the two gene activation events support this connection. Based on the current data, we do not suggest that AR may have a role in XBP-1 splicing directly.

2. *There are several points not clear in terms of methodology in this data mining exercise. How were the UPR genes selected? It would be worthwhile correlating the UPR gene signature with both hormone niave and castrate resistant prostate cancers rather than AR levels per se.*

   - The UPR gene signature from GSEA MSigDB was used in the analysis. (http://www.broadinstitute.org/gsea/msigdb/geneset_page.jsp?geneSetName=REACTOME_UNFOLDED_PROTEIN_RESPONSE). This is now included in the Materials and Methods.

   - We have done GSEA analysis with the UPR gene set in two prostate cancer cohorts (GSE6919 and GSE35988), which include CRPC tissue samples. Although there is a trend of decrease in UPR gene expression in metastatic tumors compared to primary localized hormone naïve tumors, the difference did not reach significance.

3. *AR levels were grouped into three groups. The intermediate group does not seem to be informative. What was the data if AR expression was simply considered in two groups? For panel B, for readers' ease, it would be useful if the individual p values for each of the genes highlighted to be included.*

   - As suggested by the Reviewer, we have now repeated the analysis where the AR expression was divided into two groups, instead of three. As presented in Rebuttal Figure 4 [Figure omitted upon Authors' request], the differences were still significant. We feel that presentation of the data in three groups is more detailed and informative and therefore would like to keep it in that manner in the manuscript.

   - The individual p-values for the different genes are now given in a table below the picture in Figure 2B.

4. *VCaP data - should at least be shown in supplementary rather than simply "data not shown"*
   Other clinical parameters should be included in the analysis, including tumour stage and grade. Given the expression of AR in stroma, it would be useful to have information on the

relative amount of stroma included in the samples.

We have now included the VCaP data in Figure E4A and clinical parameters of the tumor samples in Table E5. Gleason score and XBP-1S staining were also compared, but no correlation was found (data not shown). We agree with the Reviewer that it would be useful to have information on the relative stroma amount in the samples given its role in interacting with the glandular cells. However, the majority of the AR in the prostate is in the glandular cells from which the cancer arises. We therefore feel that this information will not affect the conclusions that we have derived from the current data.

5. *Supplementary Figure 1 - it is not clear what the gene list represents? What were the criteria used to generate the gene list?*

   Please see response to point 2 above.
6. *It should be made clearer that data in panels A-C were from in vitro studies while that of D-F were from xenografts.*

   - The figures have been rearranged and the experiments involving xenograft material are now labelled accordingly which made the data easier to follow now. We thank the Reviewer for the suggestion.

7. *In the vitro work, besides R1881, was there additional confirmation data using another androgen such as DHT?*

   - In experiments using DHT we essentially got the same results which are now presented in the revised manuscript (Figure E4B).

8. *Also, what is the response of the CWR22 to R1881? Is the converse true?*

   - CWR22 is a human primary prostate cancer xenograft (grows only as a xenograft) that regresses upon castration with a subsequent drop in PSA levels. However, after some time the tumor relapses into a castration resistant form (Nagabhushan et al, 1996). In this *in vivo* setting, the effect of testosterone removal is very clear (Figures 2C-E). Since R1881 releasing pellets are not available, we could not do a similar experiment. However, given the similar effects of R1881 and DHT on UPR gene expression *in vitro*, we do not expect any significant differences in this setting.

9. *JNK data is interesting, but is JNK signaling still inhibited in the IRE1 knockdown?*

   - Knockdown of IRE1α leads to a slight increase in phosphorylated JNK with no further change after 24h of TG treatment (Rebuttal Figure 5) [Figure omitted upon Authors' request], suggesting that under these conditions IRE1α does not significantly affect JNK signaling in LNCaP cells.

10. *Supplementary Figure 2 - Panel F - In the control lanes, it would appear that total JNK level was increased following R1881 treatment, despite undetectable p-JNK. There was no mention of this.*

    - This blot has been replaced by a more representative one where the main result is as before; androgens lower the amount of UV-induced JNK, however, there are no changes in total JNK levels in response to R1881. We thank the Reviewer for pointing this out.

11. *"....CHOP is directly downregulated by AR' - This reviewer is unsure if the data presented here would necessarily confirm the 'direct' nature of the regulation. The statement would suggest that CHOP is transcriptional target of AR.*

  - We agree with the Reviewer on this and have deleted this sentence.

12. *Panel F - Comparing to data shown in Figure 2C, the expression of XBP-1S, p-eIF2a and total-eIF2a (control lanes) seemed not to be consistent between the two figures.*

  - In the repeat of our time course experiments, such as those shown in these figures, the induction profiles of the same protein in different experiments can be skewed by -/+ 6-12 h. We believe that the differences that the Reviewer points out is due to this variation.

13. *Supplementary Figure 3B - CHOP level seemed to be suppressed for a long period (up to 84 hours) without any evidence of recovery. Is this not surprising?*

  - The prolonged downregulation of *CHOP* mRNA expression that we observe coincides with the continued downregulation of pPERK and p-eIF2α levels at these later time points (at least up to 72h). In contrast, in the new data that we provide, CHOP protein levels are slightly increased upon androgen treatment (Figure 2G). The mechanism(s) behind this divergent expression is not known at present: however, as global translation would be increased as a result of reduced levels of p-eIF2α, this could give rise to an increase in CHOP protein levels that we observe. This possibility is further reviewed in the Discussion.

14. *It would be good to show the data for VCaP.*

  - We have now included these data in Figure E4A.

15. *Are the ChIP data reversed by the presence of anti-andogens?*

  - We have not determined whether the ChIP data would be reversed by anti-androgens. However, as knockdown of AR leads to a significant decrease in the expression of the UPR markers, we would expect that anti-androgens would inhibit AR binding to its response elements associated with these genes. On the other hand, use of the anti-androgen MDV3100 along with knockdown of IRE1α did not provide any additional efficacy in cell growth inhibition (Figure E7E). This may suggest that there may not be any added benefit of combinatorial inhibition of the IRE1α pathway and AR as a potential therapeutic strategy.

16. *What about AR binding to XBP-1S? Is there any data on this from the ChIP?*

  - There is no AR binding in the vicinity of the XBP-1 according to the ChIP-Seq data in LNCaP cells.

17. *It is not clear how the manipulation of IRE1 and XBP1 interact with the AR status, and vice versa. Also, the use of anti-androgen would be useful here.*

  - The expression level of AR is not significantly affected by IRE1α or XBP-1 knockdown (Figure E7D). As noted above, the anti-androgen MDV3100, in combination with IRE1α knockdown, did not have a synergistic effect on cell viability (Figure E7E).

18. *The data (both in vitro and in vivo) would be strengthened by inclusion of more than one derived clone for each KD experiment.*

- We have now included more clones for shIRE1α. As shown in Figure E7C, both IRE1α knockdown clones displayed reduced colony forming ability compared to control cells.

19. *Panels E and F are effectively the same data. Panel F may be considered to be in the supplementary information.*

- We have now deleted Panel F and show only pictures of the tumor size at the time of harvest (Figure 5E).

20. *It is unclear what is the relevance to AR mediated growth in this experiment. It is highly likely that such growth suppression would be observed in AR negative cells.*

- As suggested by the Reviewer, we observe reduced growth when IRE1α or XBP-1 are knocked down in PC-3 and DU145 cells which are AR negative (Rebuttal Figure 6) [Figure omitted upon Authors' request], These data indicate that IRE1α or XBP-1 are important survival factors in both AR-positive and AR-negative PCa cell lines. However, it is unlikely that the mechanisms underlying the pro-survival function of IRE1α-XBP1 arm in AR negative cell lines would be the same as in AR positive cells. In PC-3 and DU145 cells IRE1α and XBP-1S target expression would be regulated through different signaling pathways than AR and thus their regulation and impact on growth will be different than in AR positive cells. Since this requires significant further investigation, we would prefer not to include these data in the manuscript.

21. *Is apoptosis the sole reason for difference in tumour size? Any difference in proliferation rates, perhaps assessed by Ki67 staining?*

- As suggested by the Reviewer, we now show that there is a significant decrease in the proliferative marker PCNA in sh-IRE1α and sh-XBP-1 xenograft tumors compared to Scr control (Figure 5F). This indicates that the IRE1α arm not only affects apoptosis in PCa, but also proliferation.

22. *Supplementary Figure 5 - Is the observed increase in apoptosis driven by an increase in JNK signaling? Panel D - TG should be given in full.*

- Please see the response to point 9. Thapsigargin (TG) has now been spelled out.

23. *The use of toyocamycin is interesting. Looking at the literature, it appears to be an inhibitor of rRNA processing as well as Rio 1 (Kiburu et al, Plos One 2012). It would be important to have reassurance that the observed effects were related to IRE1a.*

- This is an important point. Figure 6C shows that spliced XBP-1 is significantly decreased in the tumors from toyocamycin treated mice compared to mice treated with saline. This confirms that toyocamycin inhibited IRE1α in these tumors. With regards to the other reported effects of toyocamycin, the concentrations that we have used are much lower than what has been reported to inhibit RNA synthesis (IC_{50} 12 μM (Ri et al, 2012) vs full activity at 75 nM in our experiments (Figure E8A). In addition, we have performed *in vitro* experiments using other small molecule inhibitors of IRE1α, targeting a different moiety of the protein, that led to XBP-1 splicing and proliferation defects similar to those obtained with toyocamycin (data not shown). These data further support the notion that it is the inhibition of IRE1α that is responsible for the observed effects with toyocamycin *in vitro* and *in vivo*.

24. *Of course, the relationship to AR function would still need to be addressed.*

    - The androgen-mediated regulation of the IRE1α pathway is dependent on the AR. Upon AR knockdown, the androgen mediated induction of the IRE1α pathway is significantly decreased (Figure 3). This dependency is further confirmed by ChIP (Figure 4) showing that AR binds in the vicinity of the genes in the IRE1α pathway. Although less delineated, the PERK arm is also affected by AR knockdown (Figure 3 and Figure E3). In addition, the anti-androgen MDV3100 led to a significant decrease in cell viability when combined with knockdown of IRE1α, however, the observed effects were neither additive nor synergistic (Figure E7E). Taken together, these data indicate that AR is important for IRE1α-mediated proliferation and survival.

25. *Like Figure 5, Panel B and D duplicated data in A and C, and could be included as supplementary information.*

    - We agree with the Reviewer and have now deleted panels B and D in this figure.

What about the effects of castration?

- Although it is a very interesting experiment to combine castration with toyocamycin treatment in xenograft experiments, this will take significant additional time to perform. We would like to suggest that this is beyond the scope of the current paper.

26. *Overall, data seem to be preliminary and clinical relevance of the magnitude of the change remains unclear.*

    - We feel that in the model systems that we have utilized, the effects observed are large and fits a consistent model supported by various lines of evidence. Although the clinical relevance and applicability of our data is currently not clear, we would like to suggest that when larger cohorts become available for study, more definitive answers will be obtained in this regard. For example, the Kaplan Meier data shows that tumors with strong expression of sXBP1 relapse in 82 months compared to those with weak expression which have a median relapse time of 110 months (Rebuttal Figure 7) [Figure omitted upon Authors' request]. Although this difference did not reach significance in this cohort, it certainly warrants further exploration.

27. *Panel B - It is unclear what the p value refers to.*

    - The p-value indicates the difference between XBP-1S staining (strong and moderate) in normal vs cancer cells. This is now indicated in the respective figure legend.

28. *Panel C - It is difficult to interpret without more information on tumour grade and stage; respective PSA levels would also be good. The response may be variable within the neo-adjuvant group.*

    - We have now included this information in the revised manuscript showing clinical parameters including tumor stage, PSA levels, treatment regime etc. (Table E5).

29. *Panel D - not sure what the p value refers to. Also, is the XBP-1S level significantly different in the recurrent group?*

    - The figure has now been altered showing the results from IHC staining of XBP-1S in untreated versus NHT treated samples. The p-value is 0.00690926. XBP-1S level in the recurrent samples were not significantly different and thus these data were removed from the figure.

30. *How does XBP-1S levels correlate with AR in human disease?*

    - Unfortunately, XBP-1S expression (as opposed to XBP-1) is not included in the global gene expression datasets that are available; we thus have not been able to do this analysis. We agree with the Reviewer that this information would be very useful when available.
    What is the role for IRE1 and XBP1 loss in CaP cells that do not have AR? Is this response still present?
    - Please see the response to point 20.

Reviewer 3

1. *Abstract. "Prostate cancer (PCa) originates from a highly secretory cell type that is regulated by hormonal signals". This sentence of the Abstract is confusing. Does it refer to the prostate as secretory organ? Please clarify here and on page 4.*

    - We have now reworded this sentence to make it clearer.

2. *Page 4: "PCa originates from a highly secretory cell type that is regulated by hormonal signals, in particular androgen signaling, via the androgen receptor (AR), which is important in all phases of PCa" It would be important to mention that androgen may have a less prominent role in castration-resistant stage of prostate cancer.*

    - We have now rephrased this sentence. It is known that AR signaling remains to be important in CRPC mediated by changes to AR itself, its cofactors, signaling pathways, as well as de novo intratumoral androgen synthesis. This is underscored by the recent development of androgen synthesis or second generation AR inhibitors which have efficacy in CRPC.

3. *Page 5: "in VCaP cells, an independent androgen responsive PCa line" I would change independent with another.*

    - We have now changed the word "independent" to "another".

4. *Figure 1A: Please enlarge the fonts of gene names. They are very difficult to read.*

    - The size of the gene names is now enlarged.

5. *The description of the results presented in Figure 2 jumps from Panel A-B to D and back to see. It is not easy to follow.*

    - The order of the figures in Figure 2 in Results has now been changed.

6. *Figure 5: Panel C and D. A colony formation assay is not exactly measuring proliferation rate of cells but foci formation in monolayer. In addition these experiments are performed in serum and therefore they do not really prove that IRE1 and XBP1 are required for androgen-dependent cell growth of LNCaP cells. It is important to show results of growth curve experiments after several time points of androgen stimulation.*

    - We have now included an experiment where both sh-IRE1 and sh-XBP-1 cell lines are treated with androgen over a period of time and cell viability was measured directly in a growth curve experiment (Figure E7A-B). Consistent with the results from the colony formation assay, this experiment shows that knockdown of either IRE1α or XBP-1 significantly decreases the rate at which androgens can induce proliferation of LNCaP cells.

7. *Supplementary Figure 5D: What is TG? This assay measures cell viability not cell growth. Please correct the Y-axis to "Relative Cell Survival".*

   - We have now changed the Y-axis label to "Relative Cell Viability" and thapsigargin (TG) is now spelled out (Figure E8).

8. *Page 9 and Figure 7A. "XBP-1S protein was weakly expressed in the benign prostate, predominantly in epithelial cells, and its expression was significantly increased in PCa specimens compared to normal cells" XBP1S expression in benign prostate is not exactly weak but it is definitely enhanced in tumor tissue. I would change to "XBP-1S protein was expressed in the benign prostate, predominantly in epithelial cells, and its expression was significantly increased in PCa specimens compared to normal tissue controls".*

   - We have now changed the text as suggested by the Reviewer.

9. *Page 9: "In addition, XBP-1S expression was reduced following neoadjuvant hormone therapy, remained low in patients that responded to therapy, and increased upon PSA recurrence indicating that XBP-1S is regulated by AR in vivo and may contribute to castrate resistant disease (Figure 7C-D). These data show that the activity of the IRE1α arm of the UPR is deregulated in human PCa and may have a role in disease progression".*
   The details of the various prostate tissues are mentioned as reported in Table S1, which is instead a list of genes. Please include the specimen characteristics, which are important for the interpretation of the data presented in figure 7C and D.

   - The detailed clinical data on the samples used in this analysis are now included in Table E5.

10. *Are "recurrent" samples castration-resistant and insensitive to hormone-withdrawal therapy? If so, it would be important to better discuss the possible role of UPR genes in castration-resistant stage of prostate cancer. Is XBP staining in recurrent samples statistically different from 1-6 and 7-12 samples?*

    - In the revised manuscript the PSA recurrent bar graph has been omitted from the figure as the data obtained for CRPC samples did not reach significance in this cohort.

11. *How the authors envision a possible role of IRE in castration-resistant stage when the manuscript data support an androgen-dependent role for UPR proteins?*
    -

    - - Based on our preliminary experiments, IRE1α-XBP-1 arm might also be important for the castration-resistant prostate cancer (CRPC). In the 22rv1 cells (AR +) that were derived from a human CRPC xenograft, IRE1α and XBP-1S expression are increased upon androgen induction (Rebuttal Figure 8A) [Figure omitted upon Authors' request]. Furthermore, toyocamycin treatment was effective in decreasing cell growth of 22rv1 cells (Rebuttal Figure 8B) [Figure omitted upon Authors' request]. This is in addition to the data shown above where in vitro CRPC model cell lines that are AR-, PC3 and DU145, are inhibited for growth upon IRE1α or XBP-1S knockdown. Thus, IRE1α-XBP-1S pathway appears to be important also for CRPC. It is important to note that AR can be activated even in the castrate state in the presence of low circulating androgens. In fact, AR has been shown to be important in castration resistant prostate cancer (CRPC) where it takes on a different role than in the earlier stages to support further tumor growth (Knudsen & Scher, 2009; Schrecengost & Knudsen, 2013; Sung & Cheung, 2014).

-

References:
- Knudsen KE, Scher HI (2009) Starving the Addiction: New Opportunities for Durable Suppression of AR Signaling in Prostate Cancer. Clinical Cancer Research 15: 4792-4798

-
- Nagabhushan M, Miller CM, Pretlow TP, Giaconia JM, Edgehouse NL, Schwartz S, Kung HJ, White RWD, Gumerlock PH, Resnick MI et al (1996) CWR22: The first human prostate cancer xenograft with strongly androgen-dependent relapsed strains both in vivo and in soft agar. Cancer Research 56: 3042-3046

-
- Ri M, Tashiro E, Oikawa D, Shinjo S, Tokuda M, Yokouchi Y, Narita T, Masaki A, Ito A, Ding J et al (2012) Identification of Toyocamycin, an agent cytotoxic for multiple myeloma cells, as a potent inhibitor of ER stress-induced XBP1 mRNA splicing. Blood cancer journal 2: e79

-
- Schrecengost R, Knudsen KE (2013) Molecular Pathogenesis and Progression of Prostate Cancer. Seminars in Oncology 40: 244-258

-
- Sung YY, Cheung E (2014) Androgen receptor co-regulatory networks in castration-resistant prostate cancer. Endocrine-Related Cancer 21: R1-R11

2nd Editorial Decision 26 February 2015

Thank you for the submission of your revised manuscript to EMBO Molecular Medicine. We have now received the enclosed reports from the Reviewers that were asked to re-assess it. As you will see they are now globally supportive and I am pleased to inform you that we will be able to accept your manuscript pending the following final amendments:

1) Reviewer 1 would like you to further discuss two issues. S/he also suggests performing further experimentation. Although of course we will not be asking you to do so, should you have data that would further support the point it, please add to the manuscript. Can you please provide an additional copy of your manuscript during the submission process highlighting your changes?

2) We are now encouraging the publication of source data, particularly for electrophoretic gels and blots, with the aim of making primary data more accessible and transparent to the reader. Would you be willing to provide a PDF file per figure that contains the original, uncropped and unprocessed scans of all or at least the key gels used in the manuscript? The PDF files should be labeled with the appropriate figure/panel number, and should have molecular weight markers; further annotation may be useful but is not essential. The PDF files will be published online with the article as supplementary "Source Data" files. If you have any questions regarding this just contact me.

3) As per the checklist you provided, the manuscript must include a statement in the Materials and Methods identifying the institutional and/or licensing committee approving the experiments, including any relevant details (like how many animals were used, of which gender, at what age, which strains, if genetically modified, on which background, housing details, etc). Could you please simply add further details on gender and housing in the relevant Materials and Methods section?

4) Every published paper now includes a 'Synopsis' to further enhance discoverability. Synopses are displayed on the journal webpage and are freely accessible to all readers. They include a short standfirst (to be written by the editor) as well as 2-5 one sentence bullet points that summarise the paper (to be written by the author). Please provide the short list of bullet points that summarise the key NEW findings. The bullet points should be designed to be complementary to the abstract - i.e. not repeat the same text. We encourage inclusion of key acronyms and quantitative information. Please use the passive voice. Please attach these in a separate file or send them by email, we will incorporate them accordingly.

5) Please make sure you upload the tables as supplementary information to avoid the system merging them with the manuscript file.

I look forward to reading a new revised version of your manuscript as soon as possible and in any case within 2 weeks.

***** Reviewer's comments *****

Referee #1 (Remarks):

Remarks to the authors:
The manuscript by Sheng and Arnoldussen provides data suggesting a crostalk between androgens and UPR signaling. Results provided include in vivo and in vitro approximations, implicating the occurrence of this novel crosstalk in prostate cancer.
Most of our concerns were solved; however there is only one point still missing. This study deserved publication in EMBO Mol Med. Most data is novel and the in vivo studies clean. This study will certainly have great impact in the field.

Specific Comments:
Data presented in the manuscript suggest a regulation of androgens on the UPR signaling. The authors suggest the occurrence of IRE1 up-regulation and the down-regulation of the PERK pathway. Data provided suggest that the role of androgens on the UPR is at the level of the expression of ER stress sensors. Therefore, the effects observed by authors on UPR regulation could be a specific effect due to changes on the expression of ER stress sensors IRE1α and PERK. Can the authors discussed this point or performed additional experiments to solve go deeper into this mechanism of action?
In the point 3, we ask why there are discrepancies in the kinetics between transcriptional targets of XBP-1s and the protein levels of XBP-1s. Although the authors give 3 possibilities that might explain these discrepancies it seems that androgens have more pleiotropic effects besides the UPR and therefore have divergent effects as described by authors in figure E7A. This issue could be further discussed in the text.

Referee #3 (Remarks):

The authors have addressed my previous concerns and revised the manuscript accordingly.
