# Supplementary material for: Divergent androgen regulation of unfolded protein response pathways drives prostate cancer
Source: EMBO Mol Med. 2015 Apr 11;7(6):788–801. doi: 10.15252/emmm.201404509 (PMC4459818; doi:10.15252/emmm.201404509)
Supplement: Supplementary file 6 [file emmm0007-0788-sd6.pptx]

## Slide 1
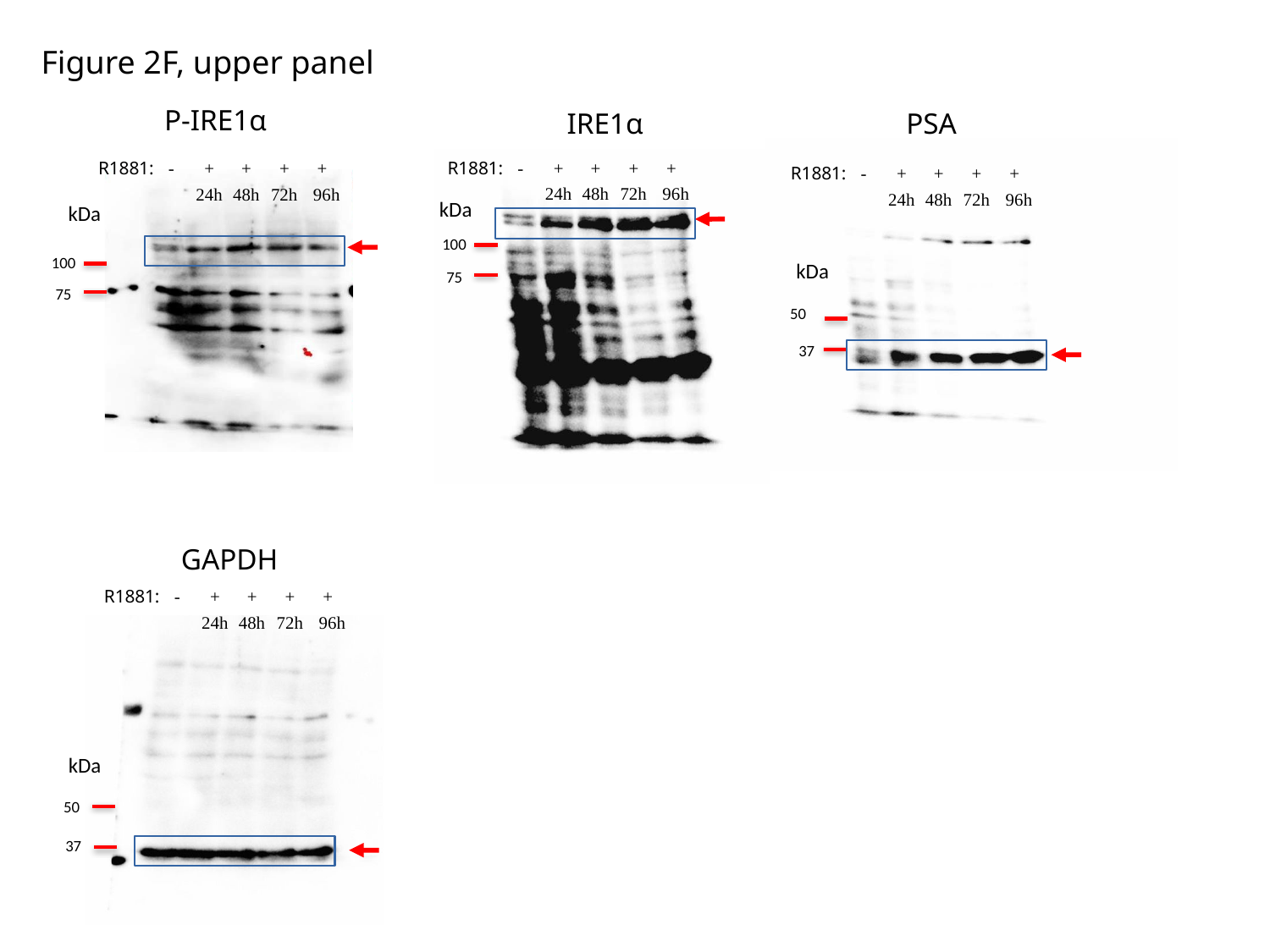

Figure 2F, upper panel
P-IRE1α
IRE1α
PSA
R1881:
-
+
+
+
+
R1881:
-
+
+
+
+
R1881:
-
+
+
+
+
24h
48h
72h
96h
24h
48h
72h
96h
24h
48h
72h
96h
kDa
kDa
100
100
kDa
75
75
50
37
GAPDH
R1881:
-
+
+
+
+
24h
48h
72h
96h
kDa
50
37

## Slide 2
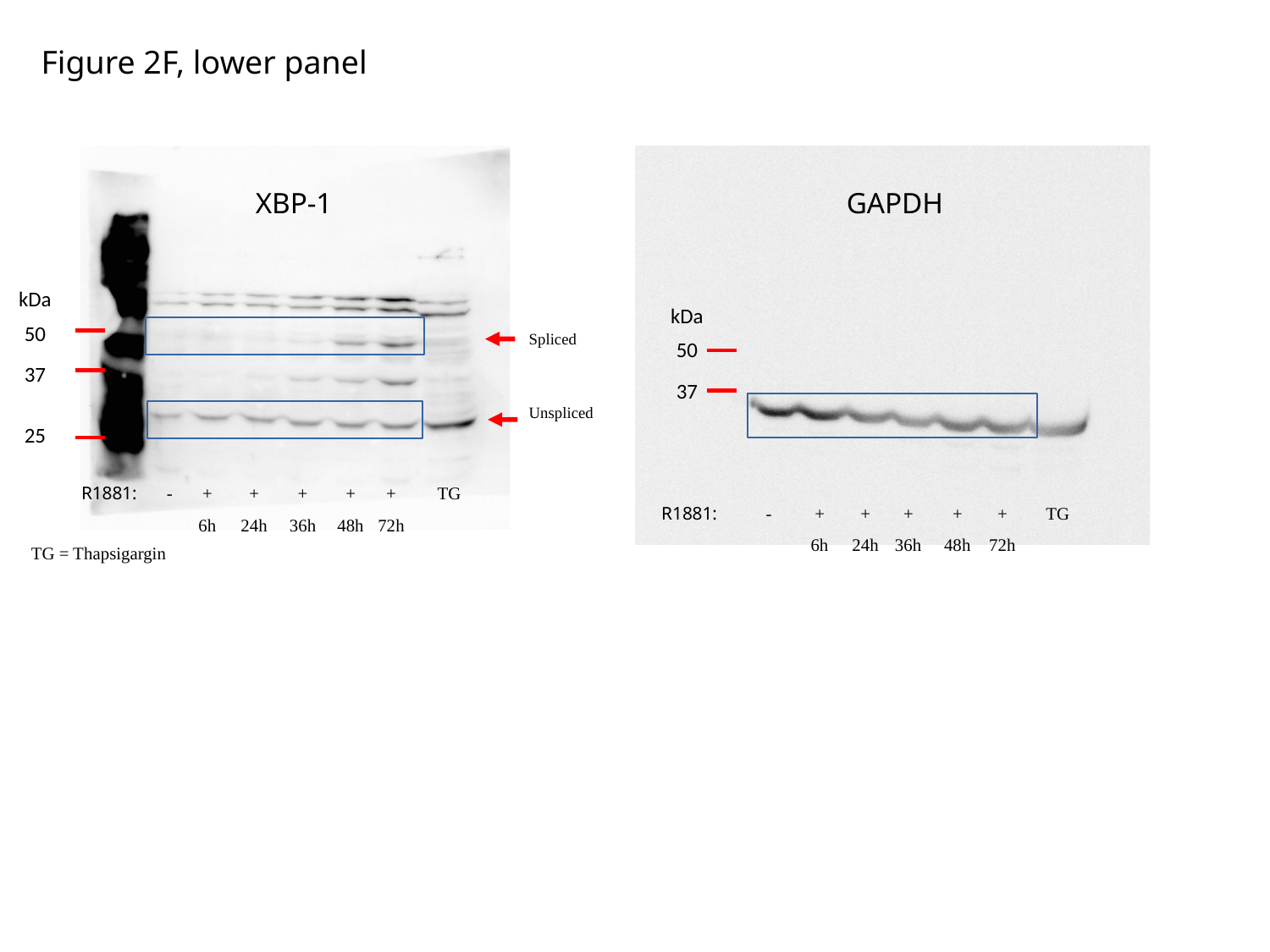

Figure 2F, lower panel
XBP-1
GAPDH
kDa
kDa
50
Spliced
50
37
37
Unspliced
25
R1881:
-
+
+
+
+
+
TG
R1881:
-
+
+
+
+
+
TG
6h
24h
36h
48h
72h
6h
24h
36h
48h
72h
TG = Thapsigargin

## Slide 3
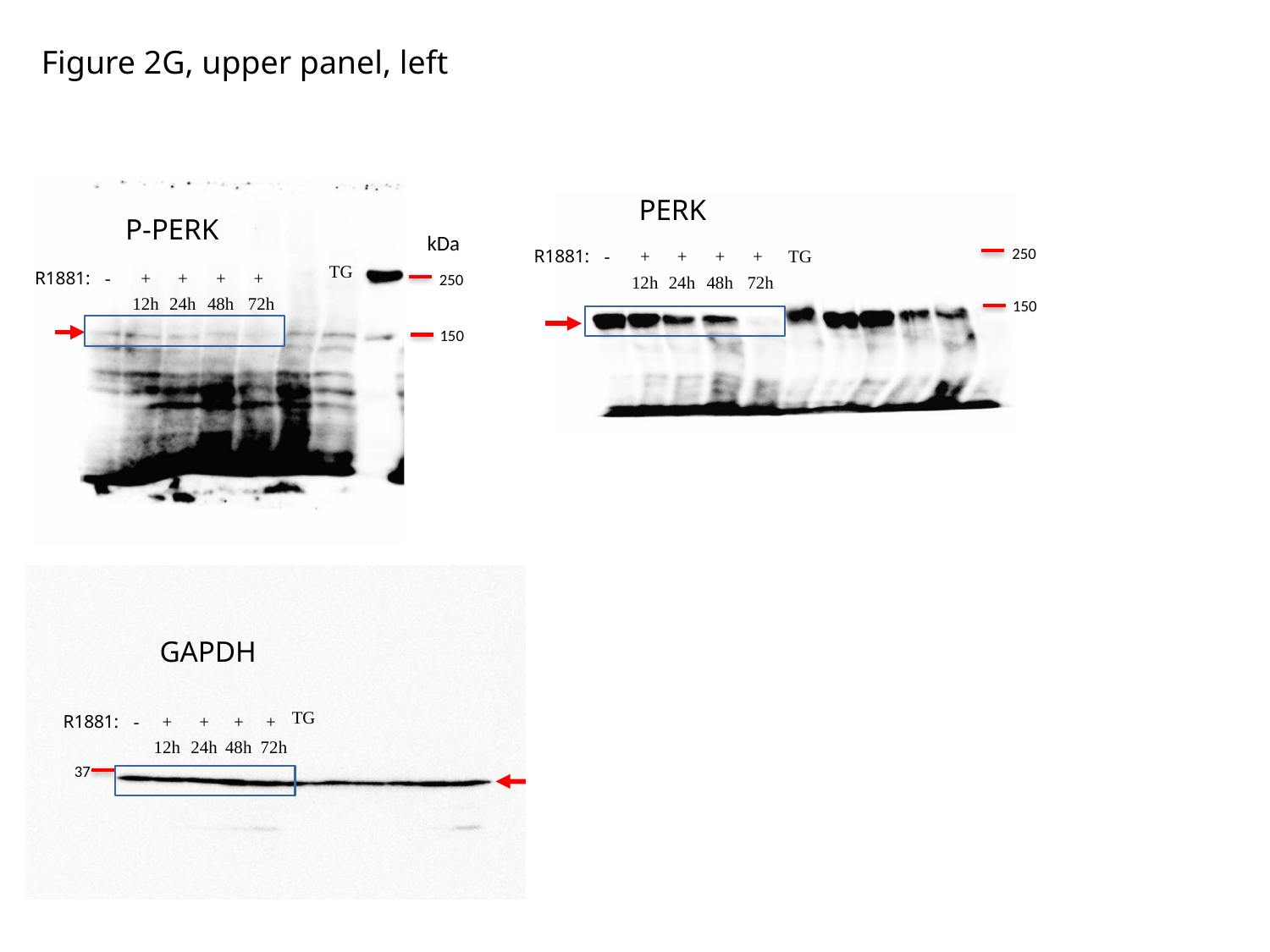

Figure 2G, upper panel, left
PERK
P-PERK
kDa
250
R1881:
-
+
+
+
+
TG
R1881:
-
+
+
+
+
TG
250
12h
24h
48h
72h
12h
24h
48h
72h
150
150
GAPDH
R1881:
-
+
+
+
+
TG
12h
24h
48h
72h
37

## Slide 4
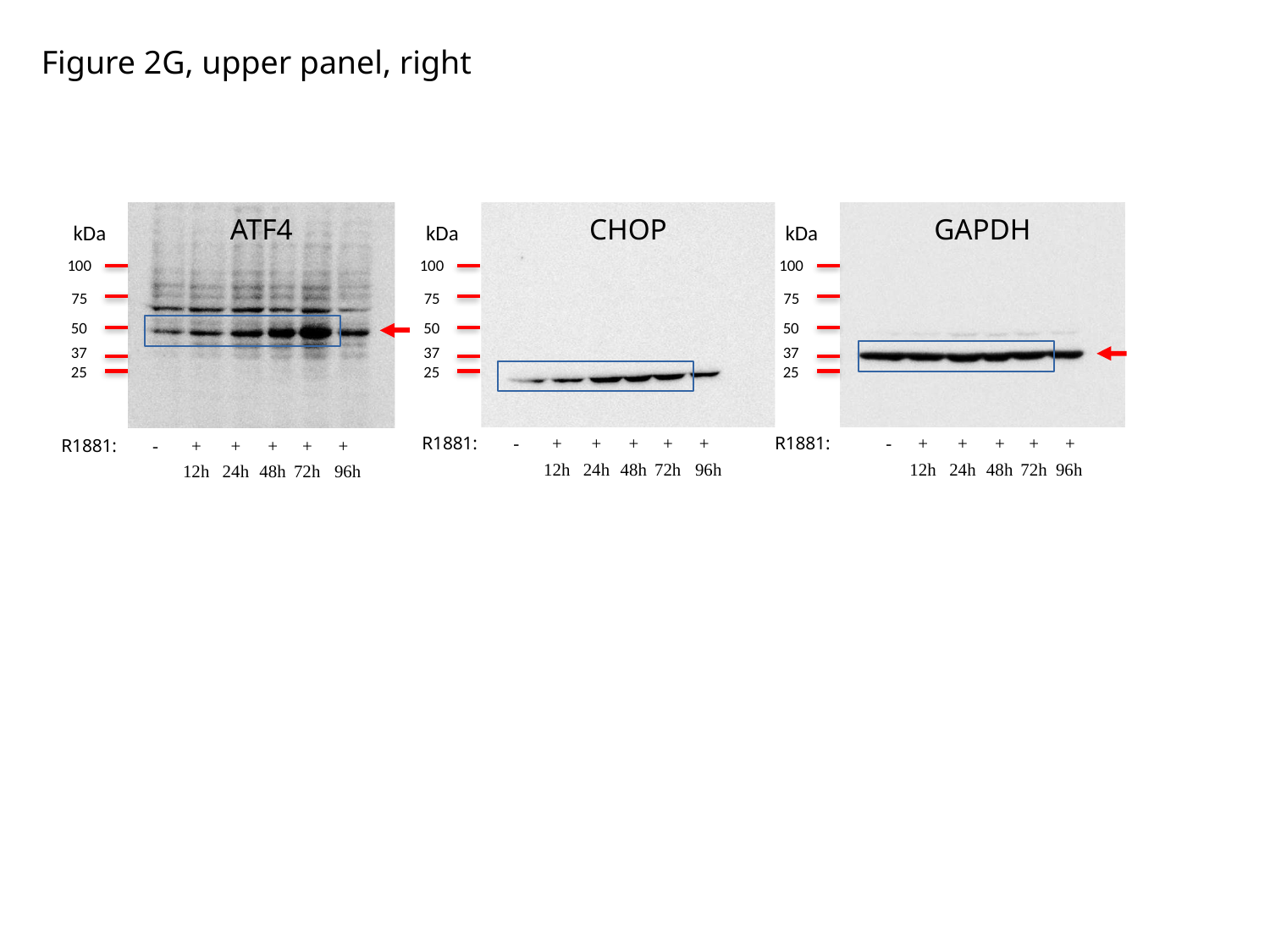

Figure 2G, upper panel, right
ATF4
CHOP
GAPDH
kDa
100
75
50
37
25
kDa
100
75
50
37
25
kDa
100
75
50
37
25
R1881:
-
+
+
+
+
+
R1881:
-
+
+
+
+
+
R1881:
-
+
+
+
+
+
12h
24h
48h
72h
96h
12h
24h
48h
72h
96h
12h
24h
48h
72h
96h

## Slide 5
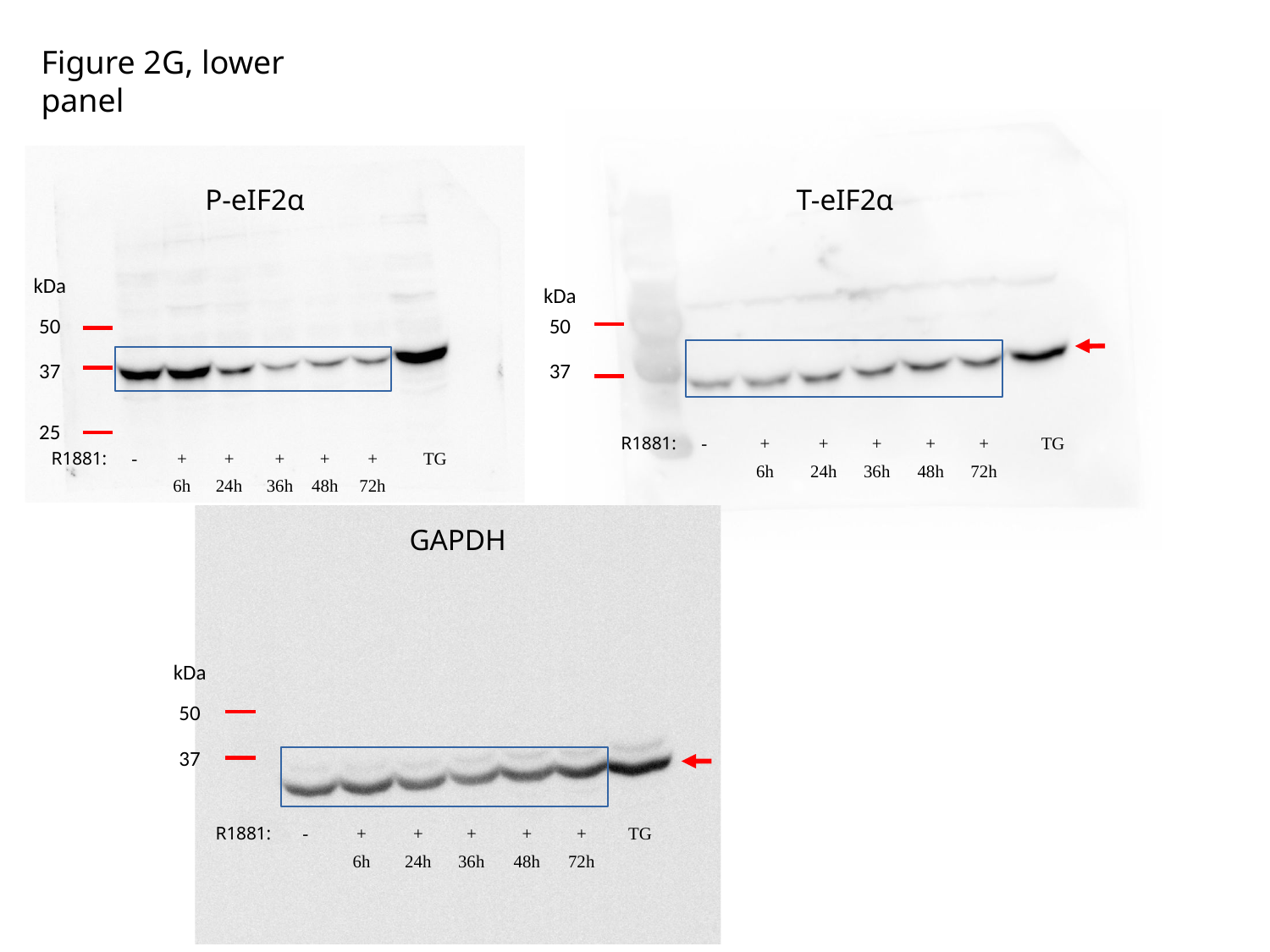

Figure 2G, lower panel
P-eIF2α
T-eIF2α
kDa
kDa
50
50
37
37
25
R1881:
-
+
+
+
+
+
TG
R1881:
-
+
+
+
+
+
TG
6h
24h
36h
48h
72h
6h
24h
36h
48h
72h
GAPDH
kDa
50
37
R1881:
-
+
+
+
+
+
TG
6h
24h
36h
48h
72h
