# Supplementary figures and images for: Divergent androgen regulation of unfolded protein response pathways drives prostate cancer
Source: EMBO Mol Med. 2015 Apr 11;7(6):788–801. doi: 10.15252/emmm.201404509 (PMC4459818; doi:10.15252/emmm.201404509)

## Slide 1
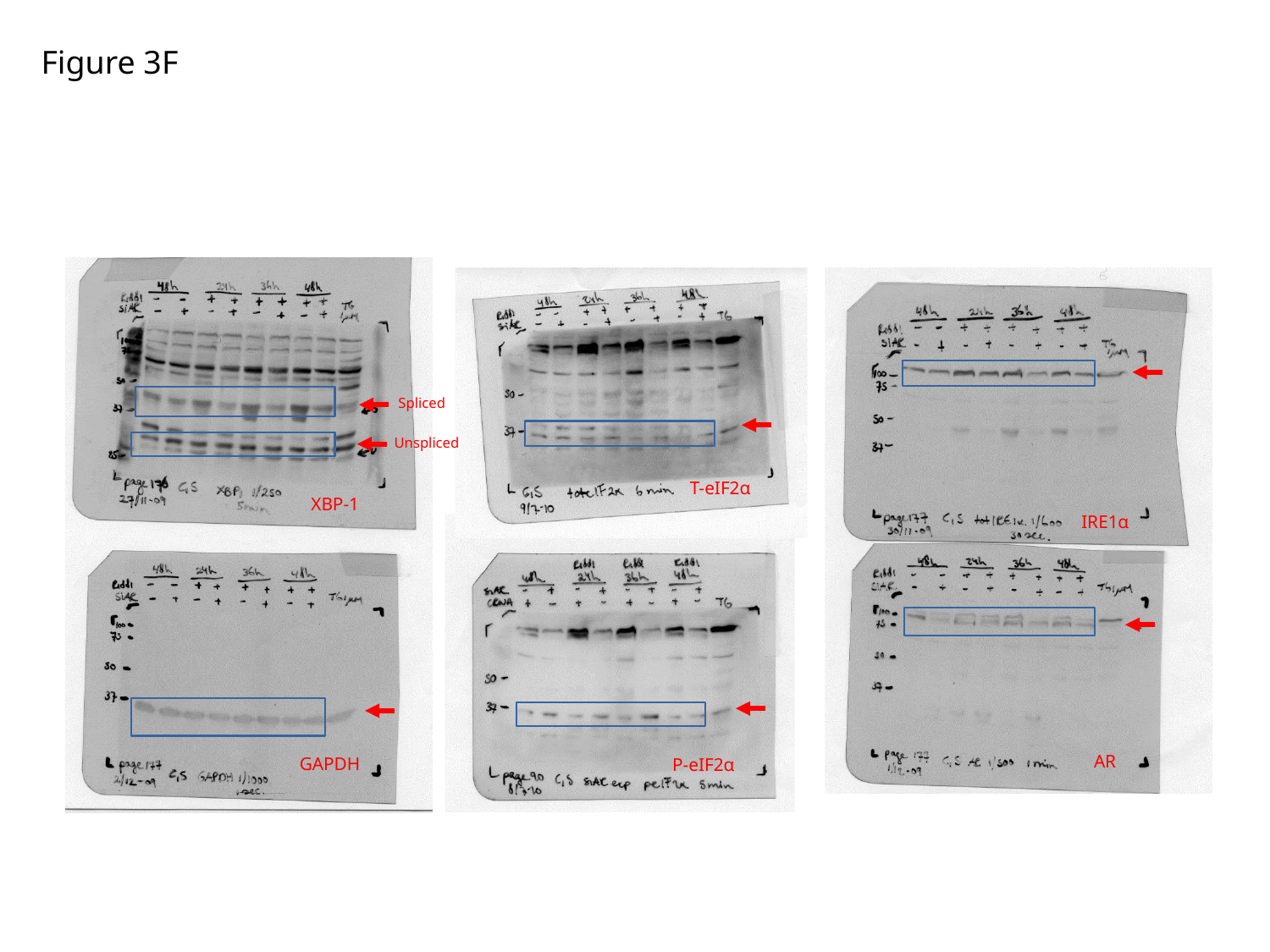

Figure 3F
Spliced
Unspliced
T-eIF2α
XBP-1
IRE1α
AR
GAPDH
P-eIF2α

Supplement: Supplementary file 7 [file emmm0007-0788-sd7.pptx]
